# Conformational dynamics of fuzzy interfaces in disordered protein complexes: mapping key residues and binding modes beyond NMR models

**DOI:** 10.3389/fbinf.2026.1907936

**Published:** 2026-09-03

**Authors:** María Laura Fernández, María Clara Bastien, Franco G. Tavolaro, Cristina Marino-Buslje

**Affiliations:** 1 Departamento de Física, Facultad de Ciencias Exactas y Naturales, Universidad de Buenos Aires, Ciudad Universitaria, Buenos Aires, Argentina; 2 CONICET - Universidad de Buenos Aires, Instituto de Física Interdisciplinaria y Aplicada (INFINA), Ciudad Universitaria, Buenos Aires, Argentina; 3 Instituto Tecnológico de Buenos Aires (ITBA). San Martín 202 CABA, Buenos Aires, Argentina; 4 Instituto Leloir/IIBBA-CONICET. Av. Patricias Argentinas 435. CABA, Buenos Aires, Argentina

**Keywords:** fuzzy complexes, fuzzy interfaces, intrinsically disordered proteins (IDPs), molecular dynamics simulations, NMR conformational ensembles, residue contact analysis

## Abstract

Understanding the dynamic behavior of fuzzy complexes formed by intrinsically disordered proteins (IDPs) is challenging due to their conformational flexibility. Nuclear Magnetic Resonance (NMR) structural bundles satisfy experimental constraints and inherently provide an overview of alternative conformations in solution. However, they assign equal weight to every sampled model, obscuring the temporal prevalence and dynamics of inter-residue contacts. In this study, we performed dual 1-microsecond (µs) Molecular Dynamics (MD) simulations for eight biologically relevant fuzzy complexes, utilizing the most distant conformers from deposited NMR models, as starting configurations. Through structural clustering and contact-persistence analysis, we classified the complexes into two distinct dynamic categories. The first group comprises complexes whose trajectories remain consistent with experimental data. Notably, large-scale motions such as relative chain rotations observed during simulations are already reflected as distinct orientations within the starting NMR conformers. Conversely, the second group comprises complexes that exhibit interaction modes absent in the structural NMR ensembles. These include alternative stable interfaces arising from chain reorientation, as well as highly dynamic interfaces characterized by rapidly exchanging contacts lacking any persistent stabilizing core. Furthermore, the microsecond trajectories captured intermittent dissociation and re-association events where the proteins sampled transient contacts through alternative regions. Overall, leveraging NMR models as a baseline for MD clustering and contact weighting, provides a systematic bioinformatics analysis to solve the atomistic details of flexible interfaces. Beyond categorizing diverse binding behaviors, this approach systematically refines the interaction surfaces across all studied cases, uncovers dynamic features that escape detection in ensemble-averaged NMR structures. Furthermore, it maps key residues to guide further experimental approaches, such as mutational studies or therapeutic targeting, among other applications.

## Introduction

Understanding the dynamic behavior and interaction patterns of protein complexes is essential for elucidating their biological functions (Wright and Dyson, 2015; Tompa and Fersht, 2009; van der Lee et al., 2014). Intrinsically disordered proteins (IDPs) introduce an additional layer of complexity due to their conformational flexibility and lack of a stable three-dimensional structure (van der Lee et al., 2014); (Sharma et al., 2015; Gsponer and Babu, 2009; Dunker et al., 2001). These proteins represent a far from negligible fraction of the protein universe, considering that they account for approximately 30%–40% of the eukaryotic proteome (Ward et al., 2004).

This structural plasticity and multiplicity of states inherently bias IDPs toward promiscuous behavior, allowing them to interact with a diverse array of molecular partners either simultaneously or across different spatial and temporal contexts (van der Lee et al., 2014). Traditionally, it was assumed that the vast conformational freedom of IDPs in solution becomes strictly restricted upon target recognition through a mechanism known as coupled folding upon binding (Wright and Dyson, 2009). However, growing evidence indicates that many disordered regions retain substantial conformational flexibility even within a bound complex (Miskei et al., 2017b).

Consequently, complexes formed by disordered proteins often lack a single, well-defined interface, displaying instead variable and transient interaction patterns. These so-called fuzzy complexes exhibit a broad range of conformations and variable contact networks that modulate their assembly and function (Sharma et al., 2015); (Hatos et al., 2022). In these systems, fuzzy regions not only preserve or enhance their structural disorder in the bound state, but this residual dynamism also directly impacts the biological activity of the complex, playing a pivotal role in the regulation and architecture of higher-order macromolecular assemblies (Wu and Fuxreiter, 2016). Despite their fuzziness, certain conformations and inter-residue contacts are more prevalent and functionally relevant within the structural bundle (Miskei et al., 2017a).

Molecular Dynamics (MD) simulations offer an atomistic view of conformational changes over time. This computational tool complements the structural information provided by NMR, which represents a set of alternative models that individually satisfy experimental data. However, these experimental models might lack other functionally relevant conformations. In this context, the analysis of major conformational clusters derived from MD simulations enables the characterization of fuzzy interfaces by identifying the most stable intermolecular interactions. This approach is particularly valuable for characterizing disordered proteins, whose function is intrinsically linked to their flexibility and ability to explore multiple conformations (Varadi and Tompa, 2015; Ghafouri et al., 2024).

While combining NMR and MD is mostly used for characterizing IDP monomers (Salvi et al., 2022; Heller et al., 2022), many approaches rely on extensive experimental datasets such as chemical shifts or relaxation data (Malm et al., 2026), which are rarely available for large assemblies. Consequently, the dynamics and contact persistence governing fuzzy interfaces within multi-protein complexes remain significantly under-explored.

In this context, combining NMR-derived models with MD simulations emerges as a robust strategy to explore the structural landscape of disordered protein complexes. By focusing on major conformational clusters, this computational framework allows for a detailed, characterization that overcomes the limits of traditional experimental methods. This approach further provides a deeper, time-resolved understanding of these dynamic systems. By identifying key interactions and prevalent conformations, our analysis provides a valuable framework for protein–protein interaction studies, particularly through mutation-based analyses. Furthermore, these structural insights establish a foundation for rational identification of therapeutic targets at the interface, among other future perspectives.

## Materials and methods

A workflow of the analysis is provided as Supplementary Figure S1 **.**

### Selection of protein complexes

We selected eight protein complexes solved by NMR from the FuzDB database (Hatos et al., 2022). The proteins that form each of the complexes (PDBs) are not fully solved experimentally, but rather there are experimentally solved segments derived from larger proteins, which often manifest significant disorder (Supplementary Figure S2). Due to this reason, we deliberately selected complexes with the following criteria: i) Ten consecutive disordered amino acids flanking the experimentally solved region. This rather subjective but strategic selection, aims to decrease the potential impact of additional amino acids that could modulate interactions by stabilizing or destabilizing specific conformations. In such a way we assume that the surroundings of the observed complex will be predominantly solvent rather than other amino acids from the same proteins. And ii) the NMR models have more than 10 models deposited in the PDB. The chosen complexes include transcription factors, splicing factors, and DNA repair among others (see Supplementary Table S1).

### Molecular dynamics simulations

We run two independent MD simulations starting from the two most distant conformers from the NMR models, by means of RMSD distance upon superposition to maximize the coverage of the experimentally observed conformational space. Using markedly different conformations as initial structures enables the identification of key interacting residues that remain consistent despite substantial differences in the starting structural state.

MD simulations were performed using GROMACS software v. 2021.5 (Van Der Spoel et al., 2005), CHARMM36m all-atom force field, TIP3P water model, and a KCl concentration of 0.15 M. While several force fields have been optimized for IDPs and experimentally validated, CHARMM36m was selected as it is a force field capable of reproducing both structured and unstructured regions (Huang et al., 2017; Robustelli et al., 2018).

The Verlet leapfrog algorithm was used with a time step of 2 fs. A cutoff of 1.2 nm was used for short-range electrostatic and Lenard-Jones interactions. Long-range electrostatic interactions were handled using the Particle Mesh Ewald method (Darden et al., 1993). The solute and solvent were coupled separately to a temperature bath of 300 K using a modified Berendsen thermostat (V-rescale) with a relaxation time of 0.1 ps (Bussi et al., 2007). The pressure coupling was fixed at 1 bar using the exponential relaxation pressure coupling (C-rescale) algorithm (Bernetti and Bussi, 2020) with an isothermal compressibility of 4.5 × 10^−5^ bar^−1^. Periodic boundary conditions were applied in all three directions. As a first step the process of energy minimization was applied to all systems. Then a first equilibration phase was performed under an NVT ensemble for 100 ps and followed by a second equilibration under an NPT ensemble for 200 ps applying position restraints on protein heavy atoms. The production runs were performed for 1 microsecond under an NPT ensemble, where the first 50 ns of the simulations were not included in the analysis (19,000 frames to be analyzed). The 1-microsecond production runs are highly computationally demanding, as disordered proteins exhibit significant mobility. Consequently, the simulation box must be large enough to allow the proteins to move freely and/or undergo extensive unfolding without reaching the box boundaries, while avoiding any undesired interaction with their periodic images.

We conducted the analysis of the results at both the chain and complex levels, using GROMACS tools: root-mean-square deviation (RMSD), and clustering over time. The method for clustering was gromos using the gmx cluster tool. Briefly, the structure with the largest number of neighbors is assigned as the representative center of the first cluster, and both the center and its neighbors are removed from the pool. This procedure is repeated iteratively for the remaining structures until all frames are classified into clusters (Daura et al., 1999). The cutoff distance was determined for each simulation such that the least populated cluster would have a single structure. This process resulted in a PBD with an ensemble of the representative structures of each cluster.

#### Residue interactions

Inter-chain contacts for each NMR model and MD ensemble of conformers were calculated with the RING server (Clementel et al., 2022; Del Conte et al., 2024). The distance thresholds to consider that two residues are in contact were set to the relaxed mode, except for van der Waals interactions that were manually set at 1.46 Å. The relaxed criterion was adopted due to the high mobility of the proteins involved in the complexes.

Contacts between chains for each set of conformers (MD1 and MD2) were represented in a contact frequency matrix. *Fij* is the contact frequency between residue i (chain A) and residue j (chain B). The *cf* is calculated as:

$$Fij=\frac{\sum_{k=1}^{N}c_{ijk}}{N}$$

where *c*_*ijk*_ is the contact state, a binary indicator variable for a cluster representative model k. If c_ijk_ = 1 the residue i and residue j are in contact in the representative model k, if c_ijk_ = 0 the residue i and residue j are not in contact in the representative model k, as reported by RING, and *N* is the total number of cluster representative models (MD1 and MD2). For the interaction analysis, we kept the contacts showing *Fij* > 0.75, in order not to have spurious contacts. The objective is to focus on contacts that remained present throughout the vast majority of the trajectory while minimizing the contribution of short-lived, transient interactions. In a second step, we expanded the analysis showing the temporal evolution of each contact discussed in the manuscript by their occupancy. Contacts were classified as: high (0.75 ≤ Fij <0.85), very high (Fij ≥0.85), or present throughout the entire trajectory (Fij = 1.0) taking a 7Å between any heavy atom cutoff. This cutoff is not universal; studies have used heavy-atom distance thresholds ranging from 4 to 10Å to define residue contacts (van Keulen and Bonvin, 2023; Xue et al., 2015; Zhang et al., 1999). We selected 7 Å because it lies within this commonly used range and encompasses all contact types defined by RING (Supplementary Material, Supplementary Appendix S1).

### Data analysis

Graphs and data analyses were performed using Python libraries (pandas, numpy, matplotlib and seaborn), on Google Colaboratory (Google colab), custom R scripts executed in RStudio (RStudio Team, 2024; R Core Team, 2025) and *ad hoc* scripts. Molecular visualization was performed using ChimeraX (Goddard et al., 2018) and Visual Molecular Dynamics (VMD) (Humphrey et al., 1996).

## Results

For this study, we chose 8 biologically relevant complexes having fuzzy interfaces (Tompa and Fuxreiter, 2008) (Supplementary Table S1). This phenomenon is prevalent when either one or both partner proteins are disordered (a brief description of the complexes’ biological function is provided in Supplementary Table S2).

For each complex, we run two MDs of 1 microsecond (1 µs) starting from the two more distant conformers of the NMR models. This is computationally challenging for intrinsically disordered proteins, due to their extended conformations. IDPs require exceptionally large solvation boxes, resulting in a high atom count that greatly increases the computational cost of microsecond-scale simulations. The MD-generated structures were clustered, revealing a wide variability in cluster population, from densely populated clusters to those containing only a single structure, underscoring the importance of clustering for elucidating underlying molecular dynamics. Recent studies have shown that clustering and weighting interactions in ensembles of IDPs or proteins with IDRs are crucial for capturing the key structural features of highly flexible proteins (González-Delgado et al., 2024).

By clustering the MD models, we were able to highlight the most persistent complex conformations and, therefore, better define the protein-protein interfaces. In a second step, we defined relevant contacts as those shared by both MD simulations.

Our results establish a hierarchy of inter-protein contacts and preferred interfaces, providing a layer of information that cannot be extracted from NMR structural models alone. While the set of NMR reported models individually satisfy experimental constraints, they assign equal weight to every sampled contact.

By adding a temporal dimension, our analysis reveals which contacts dominate the fluctuating interface and identifies interaction patterns that escape detection in the NMR structural bundle. This highlights the ability of MD to reveal the specific contacts that provide stability while simultaneously unveiling the dynamic modes that characterize fuzzy molecular recognition.

Once the results for each complex were analyzed, we grouped them into two main categories: The first group comprises proteins that remain stably bound consistent with the NMR experimental determination. Beyond stability, our analysis further characterized the core interfacial contacts, providing enhanced biological detail regarding the interaction. The second group comprises complexes exhibiting dynamic behaviors not fully captured by NMR. Including both, proteins that remained persistently bound, and proteins with intermittent interactions, in which the chains underwent transient dissociation and re-association, occasionally engaging through alternative interface regions.

### Group 1: complexes whose behaviour is consistent with the NMR experimental data (four complexes)

#### Gal11_ABD1_-Gnc4_cAD_ complex, relative chain rotation (PDB: 2LPB)

The Gcn4 central acidic domain (cAD) is an intrinsically disordered region that adopts a helical conformation upon binding to Gal11. In the NMR structure, only weak hydrophobic interactions were described, allowing multidirectional rotation of Gcn4 around Gal11 and characterizing the complex as fuzzy (Brzovic et al., 2011).

**FIGURE 1:**
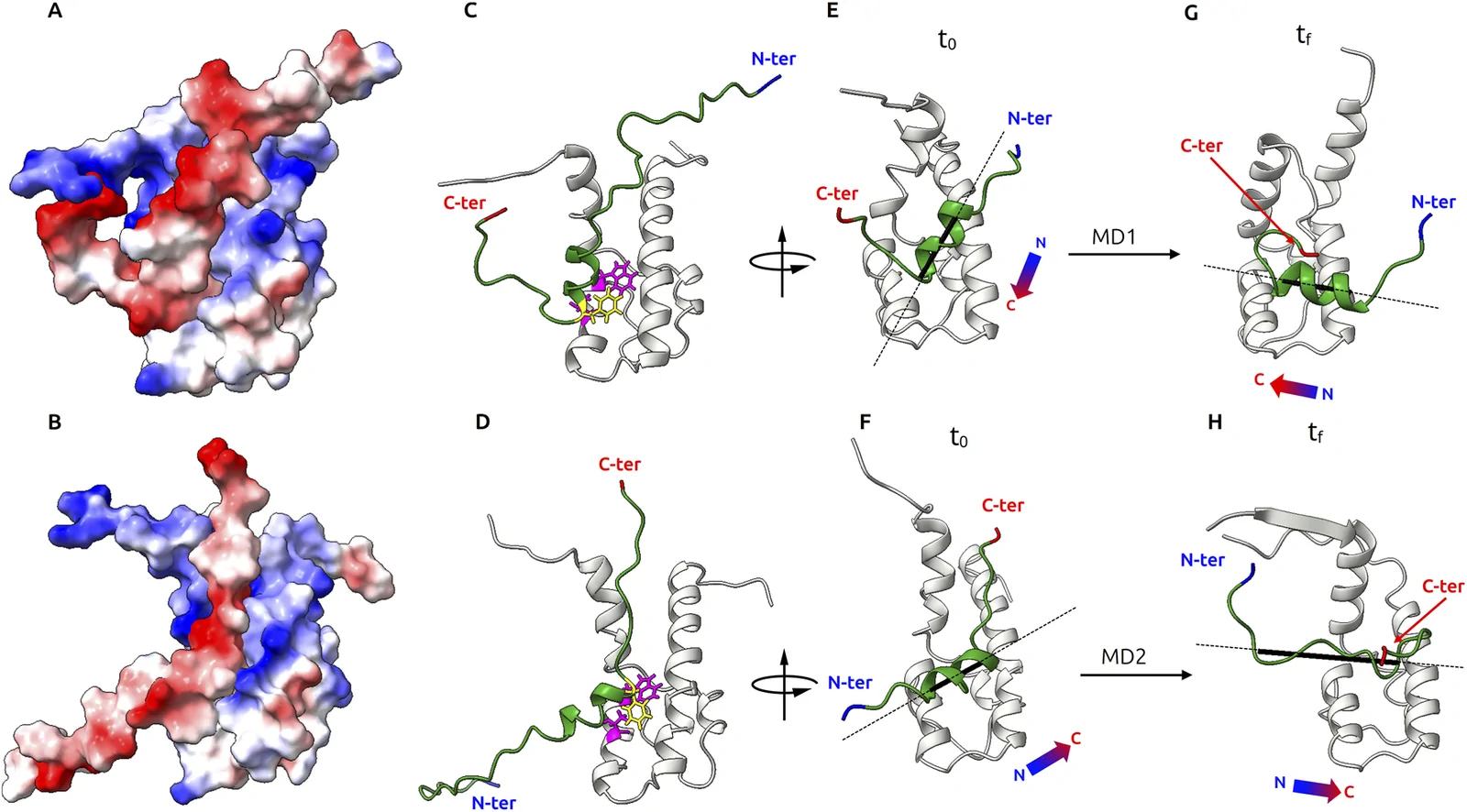
Gal11_ABD1_/Gnc4_cAD_ complex (PDB: 2LPB). The upper panel shows MD1 and lower panel MD2 results. **(A–D)** are the initial conformations taken from the NMR models. **(A,B)** are represented in surface and colored by electrostatic potential, while **(C,H)** are represented in ribbon; Gcn4 (green) and Gal11 (gray). N- and C-termini are colored blue and red, respectively, arrows indicate the N to C direction of the helix. **(C**,**D)** show the core interfacial residues as sticks (F124 of Gcn4 in yellow; W196 and L200 of Gal11 in purple). In **(E,H)** (**(E,F)**: t_0_; **(G,H)**: t_f_) the N- and C-terminal segments of Gcn4 (residues 101–112 and 131–134, respectively) are omitted and the complex is rotated for a better visualization of the helix. The dotted line indicates the axis projection of the rotating α-helix of Gcn4, arrows denote the N-to-C direction.

In our MD simulations, we used as starting points two conformers, where Gcn4 in one of them is rotated by nearly 180° relative to the other, maintaining Gal11 fixed. Remarkably, our results show that a single residue of Gcn4 (F124) maintains stable contacts with two hydrophobic residues of Gal11 (W196 and T200), interactions not previously reported. Also, the electrostatic surface analysis shows charge complementarity surrounding the binding interface, likely contributing fuzzy nature (Figures 1A,B). This contact in the Gal11 hydrophobic cleft acts as a pivot point, enabling the relative rotation of Gcn4 around Gal11. In the MD1, the system exhibited an ∼90° rotation between the initial and final conformations (t_0_ and t_f_), while the MD2 displayed a smaller net rotation between t_0_ and t_f_ but greater overall flexibility throughout the trajectory, including partial helix unfolding, in agreement with the folding upon binding reported mechanism (Figures 1E–H, Supplementary video S1; Supplementary Appendix S1).

#### SF1_NTD_/U2AF65_UHM_ complex, tight interaction throughout two interfaces (PDB: 2M0G)

**FIGURE 2:**
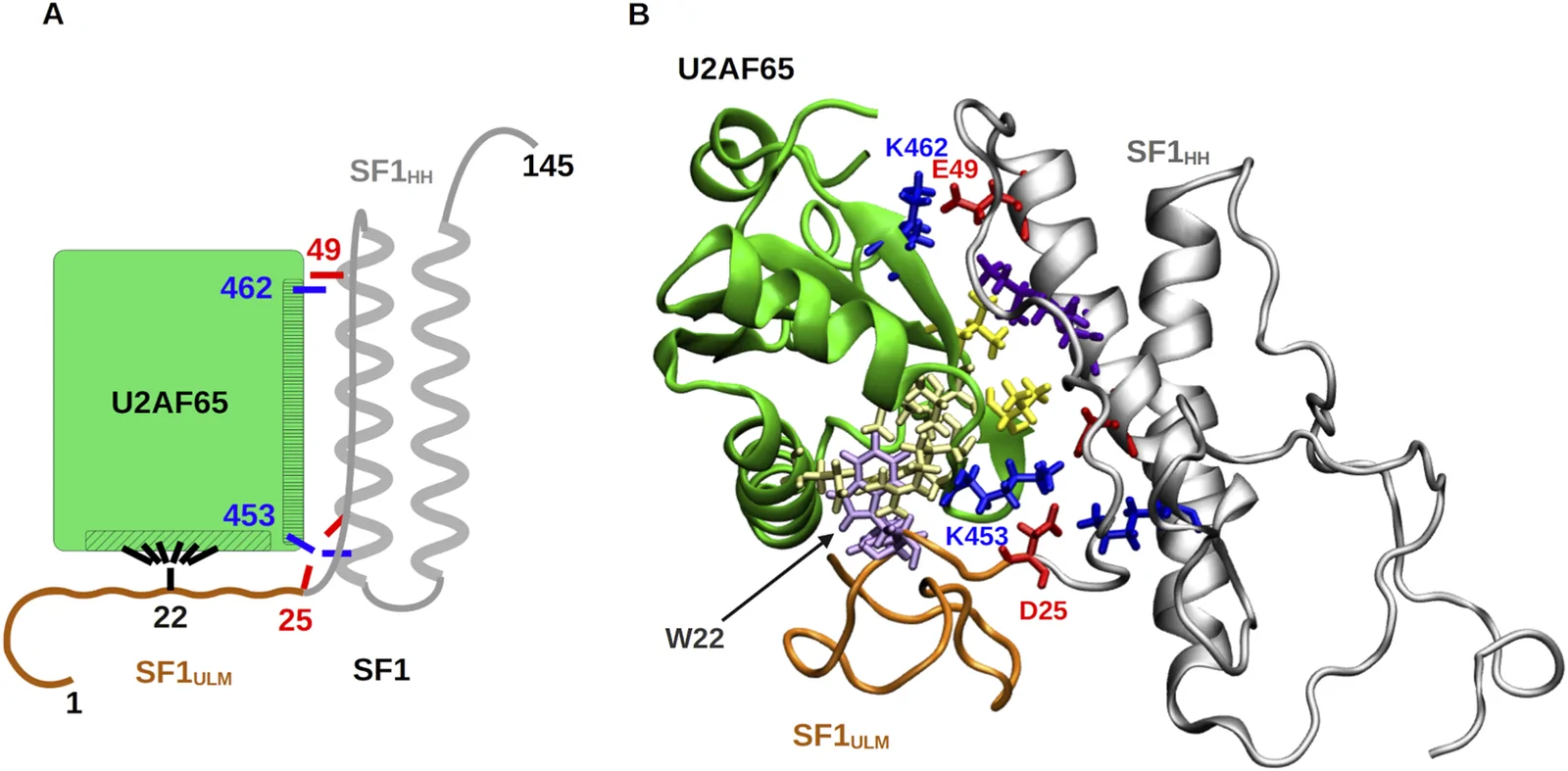
SF1_NTD_/U2AF65_UHM_ complex and core contacts (PDB: 2M0G). The representative structure of the most populated cluster (MD1) is depicted. **(A)** schematic representation of the complex, U2AF65_UHM_ (green square), the disordered region SF1_ULM_ (orange) and two-ɑ-helix hairpin motif SF1_HH_ (gray). The six contacts between W22 with U2AF65 are depicted with black lines. All the electrostatic interactions are depicted in red and blue lines for K and D respectively. The salt bridges K462-D49 and D25-k453 are indicated. **(B)** Ribbon representation with the same coloring of **(A**) the core residues are shown in sticks: SF1_ULM_ (light violet), SF1_HH_ core residues (dark violet), U2AF65_UHM_ core residues interacting with SF1_ULM_ (light yellow) and U2AF65_UHM_ core residues interacting with SF1_HH_ (yellow). W22 is pointed by a black arrow.

The N-terminal domain (NTD) of SF1 consists of two functional regions: an intrinsically disordered ULM motif (residues 1–25) and a two-ɑ-helix hairpin (HH) motif (residues 27–145); both regions interact with the C-terminal UHM domain of U2AF65 (Selenko et al., 2003; Zhang et al., 2013). Our MD simulations identified 13 core contacts: eight between U2AF65_UHM_ and the SF1_ULM_, and five between U2AF65_UHM_ and the SF1_HH_ regions. The U2AF65_UHM_/SF1_HH_ core contacts are aligned along the interface, furthermore, our simulations confirm that the K462–U2AF65_UHM_/E49–SF1_HH_ salt bridge is a persistent, fundamental interaction, capping the interface, rather than a potential contact as previously suggested (Zhang et al., 2013). We also identified an unreported closure at the other terminus, mediated by the last residue of the SF1_ULM_ disordered region (D25) and U2AF65_UHM_ K453 (Figures 2A,B, Supplementary video S2; Supplementary Appendix S1). These interactions collectively “lock” the interface, significantly refining the existing NMR-based models. Notably, the disordered ULM region remains tightly bound throughout the trajectory by six contacts mediated by W22, exhibiting higher contact stability than the structured HH region itself.

#### CBPN_CBD_-p53_TAD_ complex, tight interaction (PDB: 2L14)

**FIGURE 3:**
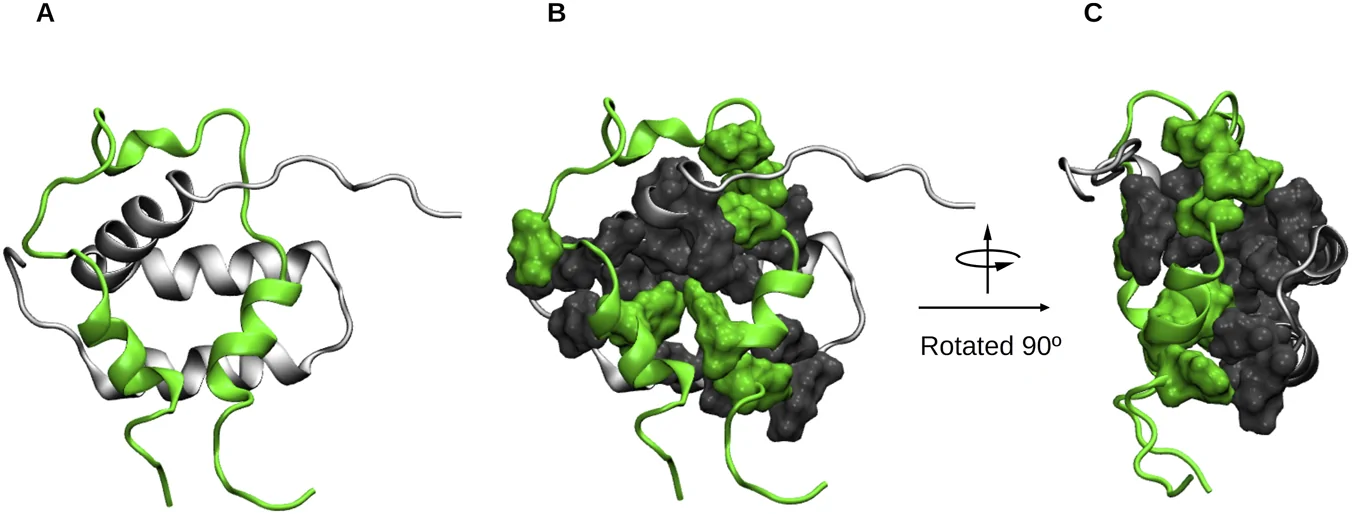
CBPN_CBD_/p53_TAD_ complex. Interacting surface and core residues visualization (PDB: 2L14), **(A)** Ribbon representation of CBPN_CBD_ (gray) and p53_TAD_ (green). p53 folded in a horseshoe-like structure around CBPN. **(B)** Ribbon representation of CBPN_CBD_ and p53_TAD_ with core residues depicted in surface. **(C)** The same complex as in panel **(B)** +90° rotated on the Z-axis. **(B,C)** show the tight association between the two proteins at the interacting surface.

This complex undergoes coupled folding upon binding between intrinsically disordered proteins (Lee et al., 2010). In this complex, p53 adopts a horseshoe-like conformation around NCBD (Figure 3). Our simulations identified several core contacts consistent with the literature, as well as interactions not previously characterized (Supplementary Appendix S1). Notably, residues within disordered regions of p53 contributed multiple persistent contacts, suggesting a stabilizing role (Supplementary video S3). Lee *et al.* identified a salt bridge between R2105 of CBPN and D49 of p53 within a predominantly hydrophobic interface. However, the D49A mutation had a minimal effect on binding affinity, suggesting the salt bridge may contribute to the specificity rather than the affinity (Lee et al., 2010). Our simulation results are consistent with this observation, while the ionic interaction remains stable in one trajectory, the salt bridge is lost at an early stage in the other, further supporting that this interaction is not critical for its stability (Supplementary video S4).

#### TFB1_PH_-p65_TAD_ complex, tight interaction (PDB: 5URN)

This complex also exhibits a disorder-to-order transition upon association, where p65 adopts an alpha-helical conformation upon binding to TFB1 (Lecoq et al., 2017). Previous studies identified multiple contacts within the ΦXXΦΦ motif (F542, L545, L546), yet mutational analyses showed that only F542 is essential for activity. Consistently, our simulations recapitulated this finding: p65 F542 emerged as the dominant interaction residue at the interface, while other contacts were less persistent. These results demonstrate that MD can independently identify functionally critical residues highlighted by mutagenesis, thereby complementing NMR data (Supplementary Figure S3, Supplementary video S5; Supplementary Appendix S1).

### Group 2: dynamic interaction patterns identified by MD simulations (four complexes)

#### 2.1 Protein-protein stable interactions (two complexes)

##### 2.1.1 TFB1_PH_/p53_TAD_ complex, relative chain rotation (PDB: 2GS0)

**FIGURE 4:**
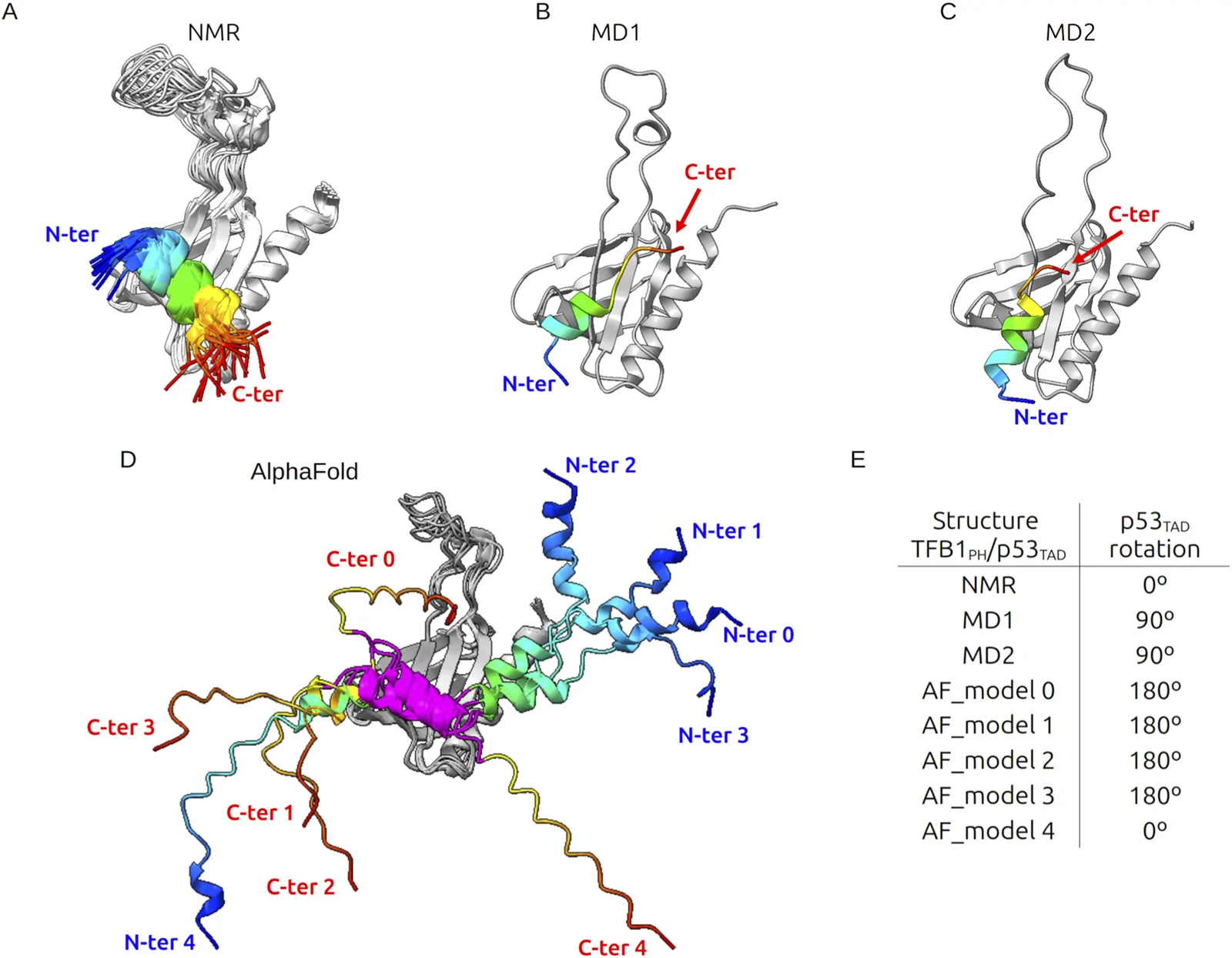
TFB1/p53 complex. **(A–C)** Ribbon representation of p53 colored in rainbow, N- to C-terminus: blue to red, and TFB1 colored in gray. **(A)** NMR models of TFB1–p53 (PDB: 2GS0). **(B,C)** Representative structures of the most populated clusters from MD1 and MD2, respectively. **(D)** AlphaFold models (0–4, cartoon representation), the α-helical region of p53 (purple) occupies the same region as in the NMR and MDs models. **(E)** Summary table showing the relative rotation of p53 in MD and AlphaFold models with respect to the NMR reference structure.

All NMR models have the same helix orientation in the interface where p53 hydrophobic residues (I50, W53, F54), and an acidic one (E56) have been identified as critical for TFB1 binding (Di Lello et al., 2006; Inga et al., 2002) (Figure 4A).

MD simulations uncovered an unreported dynamic behavior: the p53 fragment undergoes an approximately 90° rotation around a central pivot residue, F54. This alternative interface, distinct from the NMR-reported structure, remains highly stable, persisting at ∼85% of the trajectory (Figures 4B,C, Supplementary video S6; Supplementary Appendix S1).

Our simulations included only the NMR-solved segment (residues 45–58), while the experimental construct extended from residues 20–73, leaving part of p53 unsolved (Di Lello et al., 2006). To explore the possible effect of this region, we modeled the full PDB sequence using AlphaFold (Ab et al., 2024). In agreement with our results, in four of the five predicted models, p53 is rotated by ∼180° relative to the NMR orientation (Figures 4D,E, Supplementary video S7; Supplementary Appendix S1). Although static structural predictions cannot directly demonstrate dynamic fuzziness, these alternative modeled orientations provide modest, complementary support for interface flexibility and align with the rotational dynamics observed in our MD trajectories.

##### HMGB1/p53_TAD_ complex, fuzzy interface lacking persistent core contacts (PDB: 2LY4)

**FIGURE 5:**
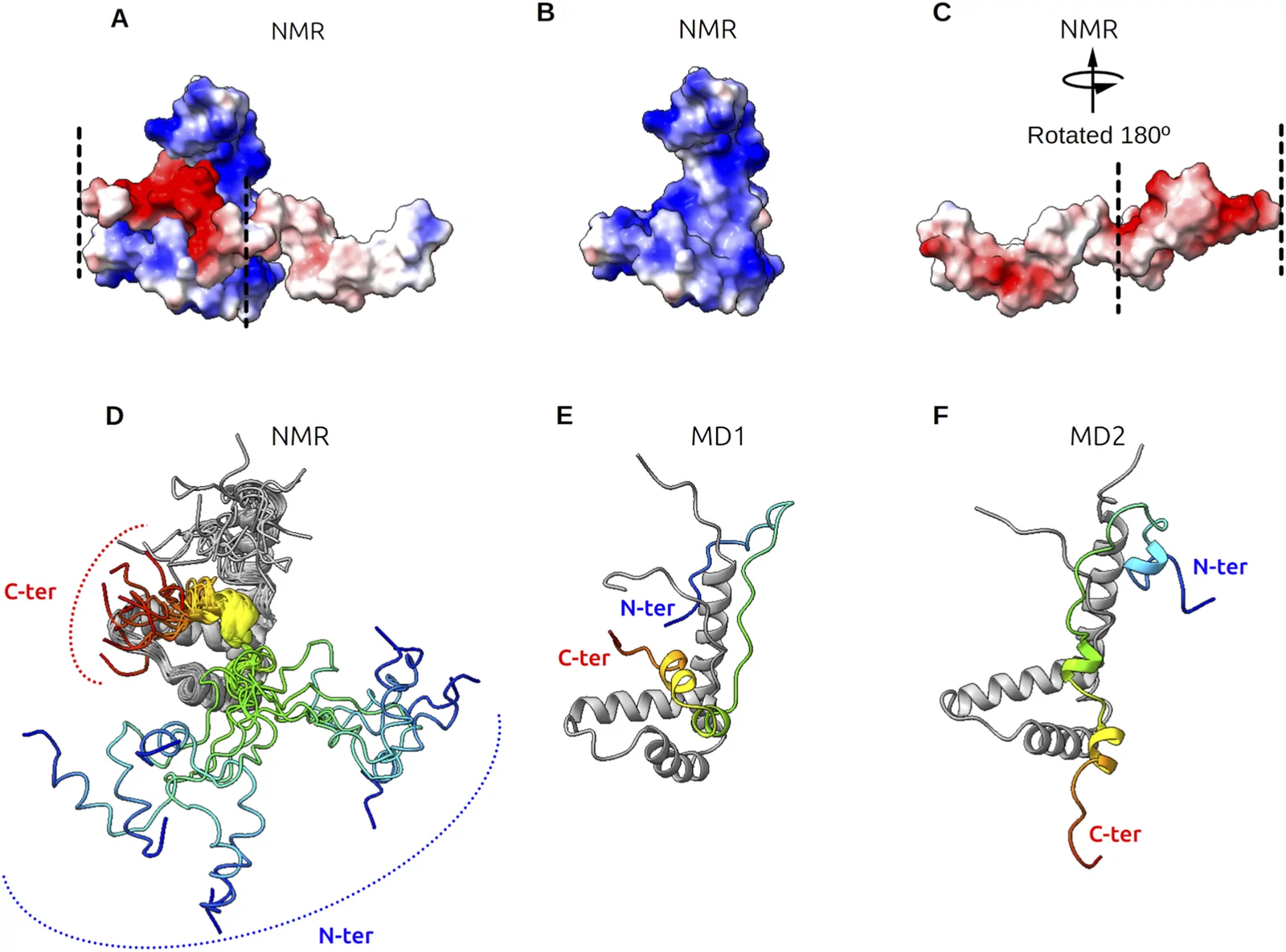
HMGB1/p53 complex, fuzzy interface and no core contacts. **(A)** NMR complex (only model 1 for clarity) both proteins are represented in surface and colored by electrostatic potential, black dashed lines indicate the TAD2 region of p53 (residues 38–60). **(B)** HMGB1 interaction surface. **(C)** p53 was rotated 180° to expose its interaction surface. **(D)** Ribbon representation of NMR models (PDB 2LY4), HMGB1 is colored in gray and p53 in rainbow from N to C terminal (blue to red). **(E,F)** Ribbon representation of representative structures of the most populated cluster from MD1 and MD2, HMGB1 is colored in gray and p53 in rainbow to facilitate the visualization of the helix position.

As observed in the TFB1PH/p53TAD complex, the HMGB1/p53TAD interaction involves the TAD2 region of p53 (residues 38–61). This region features an amphipathic helix spanning residues 47 to 54, which mediates binding with its partner via a hydrophobic interface. Figures 5A–C illustrates the nature of the interactions between the two proteins, with the electrostatic potential mapped onto the molecular surface.

The p53 helix adopts a consistent orientation relative to HMGB1 across all NMR models (Figure 5D), while our MD simulations show that, although both proteins maintain transient interactions within the same region, no stable core contacts are formed throughout the trajectories, resulting in a diffuse interface compared with the NMR models. Representative structures of the most populated clusters from both simulations are shown in Figures 5E,F. This behavior aligns with reported evidence p53 TAD2 domain engages HMGB1 primarily through short-lived hydrophobic and electrostatic interactions (Rowell et al., 2012). Across both trajectories, the interface is dominated by transient hydrophobic interactions, complemented by peripheral electrostatic contacts (Supplementary videos S8,S9).

Overall, our simulations capture a highly dynamic, fuzzy binding mode characterized by rapidly exchanging hydrophobic and electrostatic contacts, explaining the absence of persistent core interactions and highlighting a previously unappreciated level of conformational plasticity in this complex.

#### 2.2- Intermittently interacting complexes (two complexes)

##### SUMO-1/PIASX-P complex, occasionally dissociation and re-association (PDB: 2ASQ)

**FIGURE 6:**
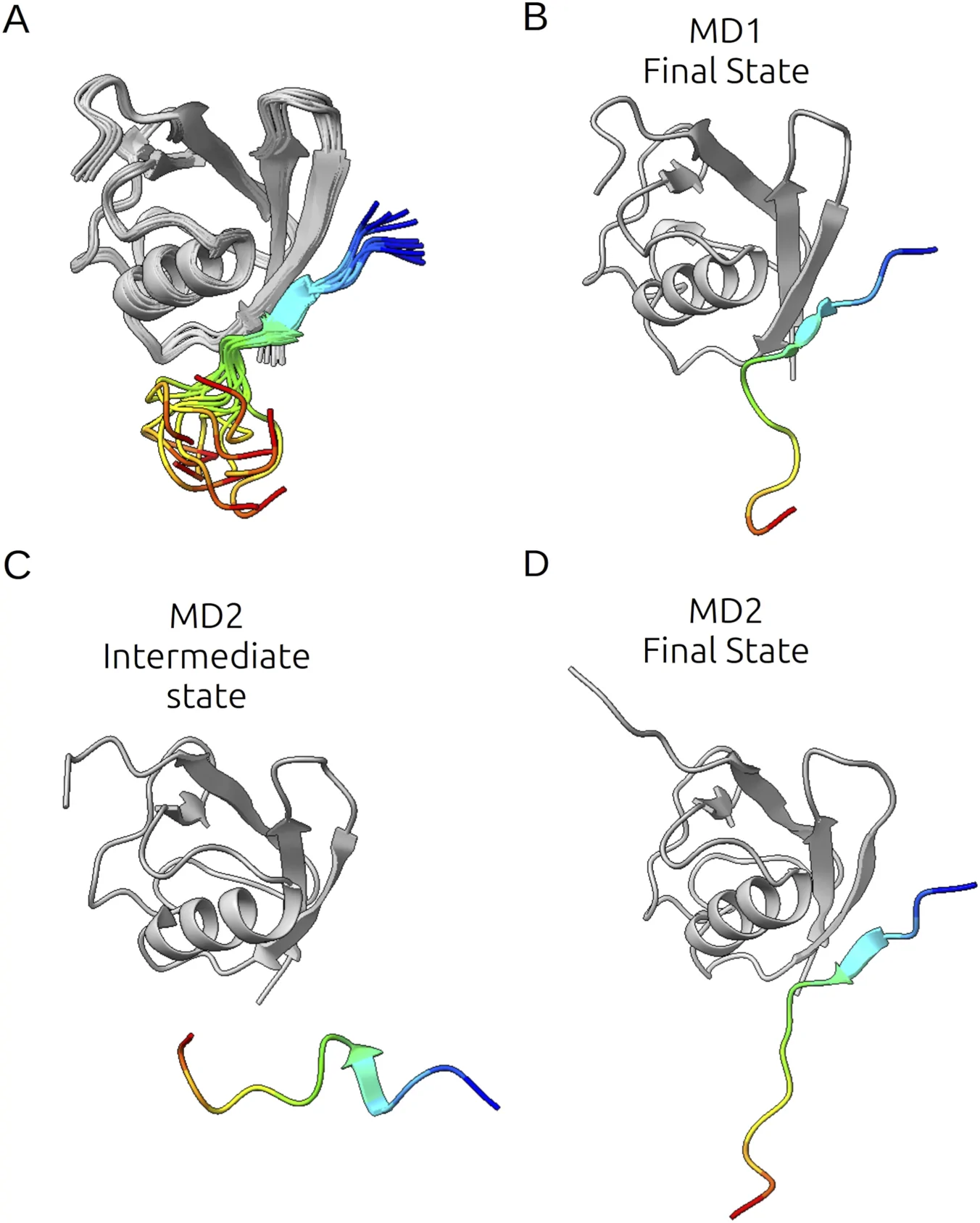
SUMO-1/PIASX-P complex. **(A)** Ribbon representation of the complex determined by NMR (PDB 2ASQ). SUMO-1 is colored in gray and PIASX-P in rainbow. **(B)** Last structure of MD1 simulation. **(C)** Intermediate snapshot of MD2 provided as an example of the complex dissociation. **(D)** Final state of the MD2 simulation.

In the NMR structure of the SUMO-1/PIASX-P complex, the PIASX-P peptide adopts a short β-strand that extends the main antiparallel β-sheet of SUMO-1 in a parallel orientation. Mutational analyses identified residues V4, I5, and L7 of PIASX-P as essential for binding (Song et al., 2005). In our simulations, the most populated clusters revealed 17 persistent core contacts stabilizing the interface, positioning both proteins similarly to the NMR models. These observations indicate that the complex predominantly adopts a stable bound conformation. However, inspection of the individual trajectories revealed differences between replicas: In one simulation the complex remained associated throughout the trajectory, while in the other, transiently dissociates early in the trajectory before rebinding through the same interface, remaining stable for the rest of the simulation (Figure 6). This behavior underscores a conformational and dynamic flexibility, despite the high number of core contacts detected (Figure 6, Supplementary video S10; Supplementary Appendix S1).

##### Transient SAE2/UBC9 interactions without core contacts (PDB: 2PX9)

**FIGURE 7:**
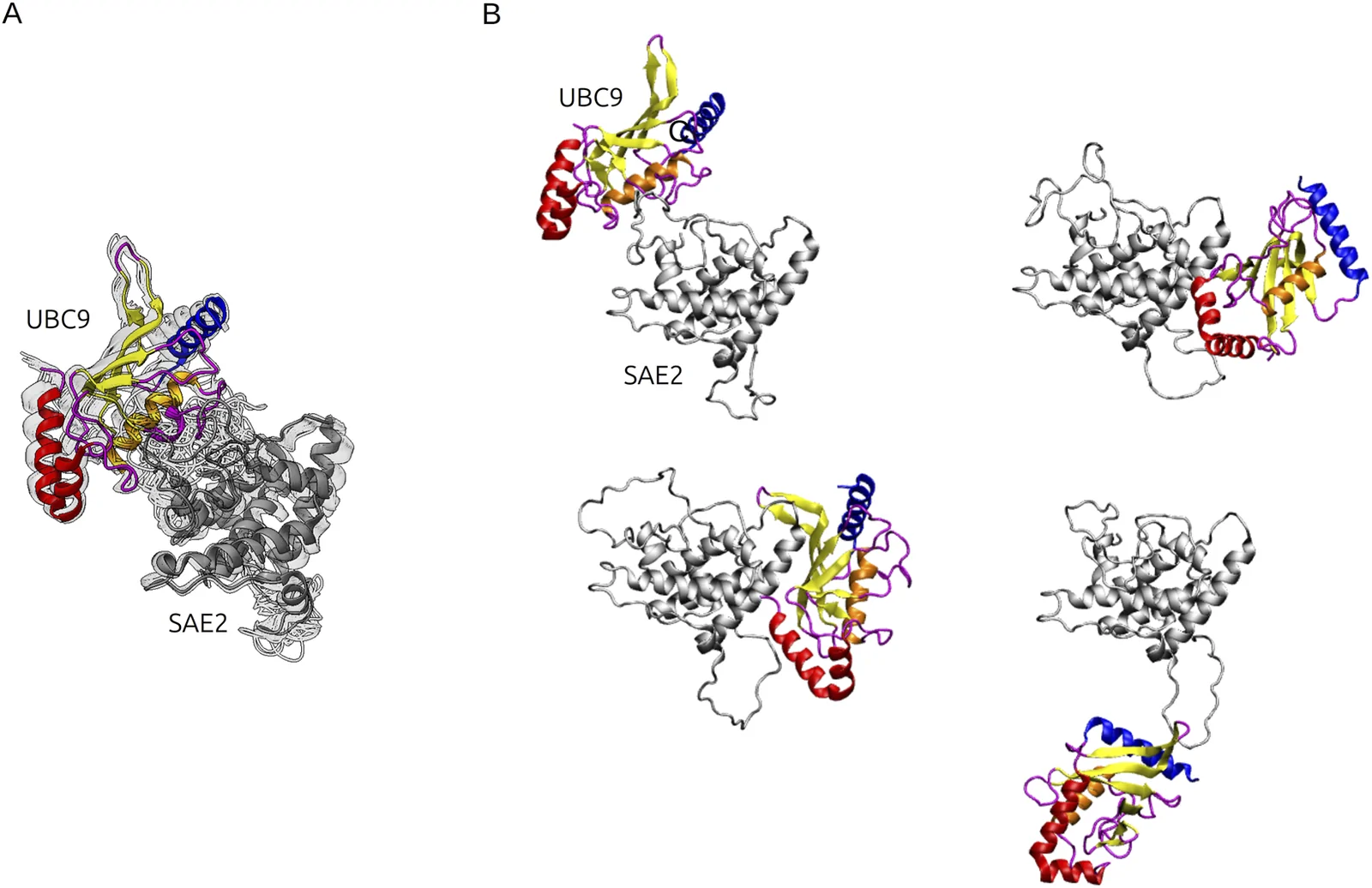
SAE2 cys domain/UBC9 complex. Ribbon representation of the complex, SAE2 Cys domain is colored in gray and UBC9 α-helices are colored blue, orange, and red; β-sheets are shown in yellow; and disordered regions in magenta. **(A)** NMR determined models (PDB 2PX9), where model 1 is depicted in color and models 2–20 are shown in transparency. **(B)** Ribbon representation of the complex where SAE2 Cys domain is shown in a fixed position in each of the four selected snapshots from the MD2 simulation. The color scheme used for UBC9 is intended to facilitate visualization of the different interacting regions throughout the simulation.

The Cys-domain of the SUMO-activating enzyme subunit 2 (SAE2) and the SUMO-conjugating enzyme UBC9 are connected through weak protein–protein interactions (Wang et al., 2007). The 20 available models solved by NMR are nearly identical and trapped in the same conformation showing a single interaction site (Figure 7A). In contrast, our MD simulations reveal that the two proteins undergo continuous dissociation and re-association events, engaging through different regions. Although these alternative interfaces should be experimentally validated, the simulations show that the proteins remain associated for most of the trajectory. This dynamic behavior is illustrated by snapshots from the simulation, shown in Figure 7B. Interestingly, SAE2 interacts primarily through its disordered regions. This behavior can also be appreciated in Supplementary video S11, where both simulations were run independently and are displayed side by side for simultaneous visualization.

## Discussion

X-ray crystallography, cryo-electron microscopy (cryo-EM), and nuclear magnetic resonance (NMR) are the primary techniques for determining high-resolution biomolecular structures. Among these, NMR is particularly valuable for characterizing conformational ensembles in solution, providing insights into the distribution, stability, and physicochemical factors governing structural interconversion (Wieske et al., 2023). However, a key limitation of NMR ensembles is their equal weighting of conformers, which does not necessarily reflect their temporal prevalence. Furthermore, since individual conformers within an NMR ensemble are refined to satisfy the same set of experimental restraints, the resulting models generally exhibit high structural similarity and remain tightly clustered. Consequently, NMR rarely captures divergent conformers or outlier states far from the main group, particularly evident in flexible proteins. This experimental approach, while highly informative, ultimately captures limited snapshots of dynamic molecular systems.

Although the conformational landscape of intrinsically disordered proteins is vast and longer simulations could theoretically reveal additional conformational states, the 1 μs trajectories employed in this study were sufficient to capture a diverse repertoire of dynamic behaviors characteristic of fuzzy protein–protein interfaces. Within this timeframe, we observed substantial conformational changes that extend beyond the resolution of NMR structural bundles, including large-scale rotational motions such as the approximately 90° rotations around central pivot residues in the Gal11–Gcn4 and TFB1–p53 complexes. Furthermore, in complexes such as SUMO-1/PIASX-P and SAE2/UBC9, the simulations revealed transient dissociation followed by reassociation events. Together, these observations indicate that the simulated timescale was sufficient to reveal a broad spectrum of interface dynamics and the temporal persistence of intermolecular contacts.

While our findings underscore the necessity of incorporating conformational flexibility to complement experimental structures, several limitations should be acknowledged. The selection of the initial conformations may introduce bias, as different members of the experimental ensemble could potentially explore alternative dynamical pathways. In addition, defining representative conformations necessarily simplifies the underlying conformational heterogeneity. Finally, complete sampling of the conformational landscape of intrinsically disordered proteins cannot be guaranteed by any practical atomistic simulation length. As discussed in Grossfield *et al.* (Grossfield et al., 2018), the adequacy of conformational sampling depends on the structural features being investigated rather than on exhaustive exploration of all possible conformations.

The analysis of the interaction dynamics provides a refined definition of the binding interface, offering a versatile framework for downstream applications. By delineating specific residues and regions critical for complex stability, our analysis could guide further experimental design, such as rational mutational studies to validate functional hot spots or explore evolutionary conservation, among other possible applications. Beyond basic biochemistry, these insights have direct implications for translational science, enabling the precise targeting of protein–protein interactions. In this context, this strategy may assist in the rational design of small molecules or peptidomimetics capable of modulating specific interfaces, targeting both highly transient dynamics and unexpectedly stable interactions that often escape traditional biophysical detection.

In conclusion, our study highlights the value of integrating MD simulations with experimental structural data to decode dynamic recognition interfaces. By capturing temporal variations and structural transitions, this framework provides a functionally relevant extension to limited ensemble representations.

## Data Availability

The raw data supporting the conclusions of this article will be made available by the authors, without undue reservation.
