# Supplementary material for: Conformational dynamics of fuzzy interfaces in disordered protein complexes: mapping key residues and binding modes beyond NMR models
Source: Front Bioinform. 2026 Sep 3;6:1907936. doi: 10.3389/fbinf.2026.1907936 (PMC13582614; doi:10.3389/fbinf.2026.1907936)
Supplement: Supplementary file 1 [file DataSheet1.pdf]

Conformational Dynamics of Fuzzy Interfaces in Disordered Protein Complexes: Mapping Key Residues and Binding Modes Beyond NMR Models.

María Laura Fernández, María Clara Bastien, Franco G. Tavolaro & Cristina Marino-Buslje.

SUPPLEMENTARY INFORMATION

Supplementary Table 1 summarizes the complexes analyzed in this study.

PDB	N°conf	Complex	Chain - source Length	length (PDB)	Full length (Uniprot)	Uniprot code
**2LPB**	13	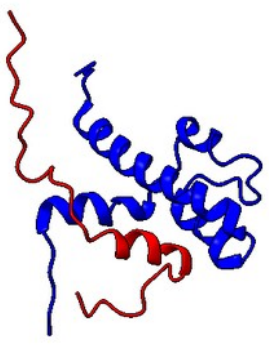	A: GAL11 yeast 158-238	81	1081	P19659
			B: GCN4 yeast 101-134	34	281	P03069
**2L14**	20	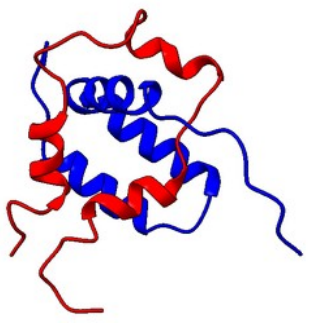	A: CBP$_{NCBD}$ mouse 2059-2117	59	2441	P45481
			B: p53$_{TAD}$ human 13-61	49	393	P04637
**5URN**	20	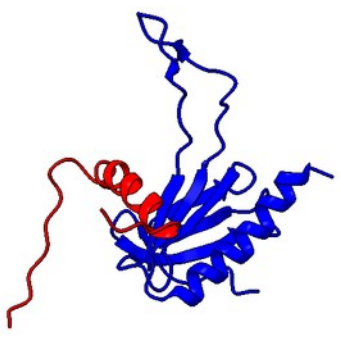	A: TFB1$_{PH}$ yeast 2-115	115	642	P32776
			B:TF65$_{TAD1}$ human 521-551	33	551	Q04206
**2M0G**	10	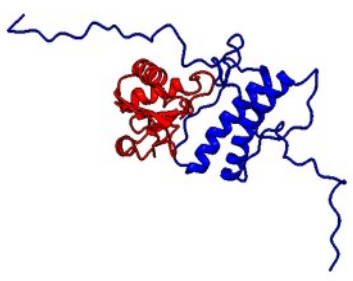	A: SF1$_{NTD}$ human 1-145	145	639	Q15637
			B: U2AF2$_{UHM}$ human 372-475	104	475	P26368
**2GS0**	20	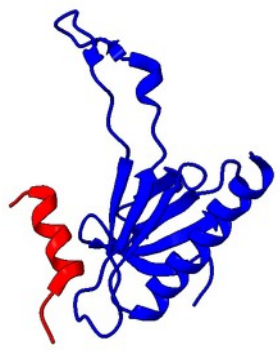	A: TFB1$_{PH}$ yeast 1-115	115	642	P32776
			B: p53$_{TAD}$ human 45-58	13	393	P04637

**2LY4**	10	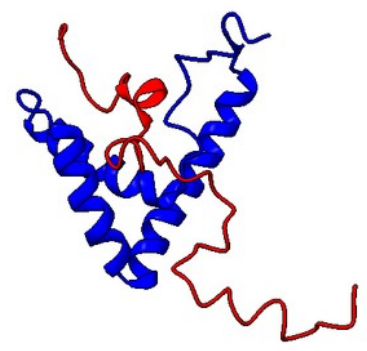	A: HMGB1 human 2-84	83	215	P09429
			B: $p53_{TAD}$ human 14-60	46	393	P04637
**2ASQ**	10	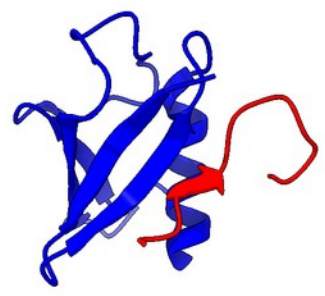	A: SUMO1 human 1-97	97	101	P63165
			B: PIASX-P human 466-488	25	621	O75928
**2PX9**	20	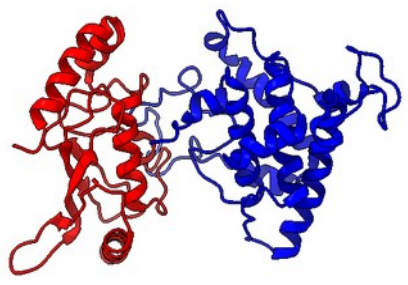	A: SUMO-AE2 human 166-382	217	640	Q9UBT2
			B: SUMO-UBC9 human 1-158	158	158	P63279

Supplementary Table 1: Selected NMR complexes (PDB code), protein names and solved region, chain length and Uniprot code. For each NMR complex, the first conformer is shown, with chain A in blue and chain B in red.

Supplementary Table 2: Brief description of the complexes biological functions

Complex PDB	Biological function
Activator-binding domain of the Mediator subunit Gal11/Med15 and acidic transcription activator Gcn4 PDB:2LPB	The activator-binding domain of the Mediator subunit Gal11/Med15 interacts with transcriptional activators to recruit RNA polymerase II and stimulate transcription<br>During nutrient starvation Gcn4 recruits the Mediator complex and the RNA Pol II to activate transcription.[1]
p53 transactivation domain (TAD) and the nuclear coactivator binding domain (NCBD) of CREB-binding protein (CBP) PDB:2L14	p53 is a transcriptional activator that mediates the cellular response to stress, participates in cell growth arrest, senescence, apoptosis, and prevents tumor formation. Inactivation of p53 is implicated in around 50% of human cancers. Its activity is mediated by the acidic TAD, divided into TAD1 and TAD2, both of which independently activate transcription [2]<br>CBP acts as a scaffold for assembling the transcriptional machinery and modifies chromatin and transcription factors. CBP is essential for p53-mediated transcription activation and for stabilizing the p53–DNA complex.[3]
Pleckstrin homology	The transcription factor B subunit 1 (TFB1) is the yeast homolog of human p62 and is one of the ten subunits of the general

(PH) domain of the TFB1 subunit of TFIIH and the acidic transactivation domain (TAD) of p65<br>PDB: 5URN	transcription and DNA repair complex TFIIH, which is essential for the initiation of the RNA polymerase II activity.<br><br>The p65 protein is a member of the NF-κB family of transcriptional regulators that control genes involved in various processes, including immune responses, cell proliferation, and survival. The TAD is crucial for mediating protein interactions with transcription factors, including TFIIH [4]
N-terminal domain of splicing factor 1 ($SF1_{NTD}$) and the C-terminal homology motif domain of U2 snRNP auxiliary factor 65-kDa subunit ($U2AF65_{UHM}$)<br>PDB: 2M0G	This complex mediates both protein–protein and protein–RNA interactions, thereby facilitating the cooperative binding of RNA during the assembly of the spliceosome [5]
The amino-terminal transactivation domain (TAD) of p53 and the pleckstrin homology (PH) domain of the Tfb1 subunit of TFIIH<br>PDB: 2GS0	The Tfb1 function has been reported in the context of the 5URN complex.<br>The p53 function has been reported in the context of the 2L14 complex.<br>The interaction between Tfb1 and the TAD of p53 is crucial for recruiting p53 to the TFIIH complex (humans and yeast). [6]
The high mobility group B1 (HMGB1) and the amino-terminal transactivation domain (TAD) of p53<br>PDB: 2LY4	HMGB1 are nuclear proteins that bind and bend DNA in a sequence-independent manner. Through its ability to bend DNA, HMGB1 facilitates de DNA binding of transcription factors, including p53.<br>The p53 function has been reported in the context of the 2L14 complex<br>HMGB1 may play a dual function in interacting with p53, both by recruiting it or stabilizing its binding to DNA, and by bending the DNA toward p53. [7]
Small Ubiquitin-like Modifier-1 (SUMO-1) and a peptide containing the SUMO binding amino acid sequence motif (SBM) derived from the protein PIASX-P<br>PDB: 2ASQ	SUMOylation is a key regulatory mechanism that controls a broad range of cellular processes, including gene transcription, subnuclear structure formation, viral infection, and cell cycle progression.<br>Since SUMO proteins appear to provide a platform for protein binding, identifying SUMO-binding motifs (SBMs) is crucial for elucidating sumoylation-dependent protein interaction networks. [8]

SUMO E2 and the Cys domain of SUMO E1PDB: 2PX9	Although ubiquitin-like proteins (Ublps) perform a variety of functions, they share a common biochemical pathway for conjugation. The process begins with an E1 enzyme, which catalyzes adenylation of the Ublp C-terminal carboxyl group, followed by formation of a thioester bond with the catalytic cysteine of E1. The Ublp is then transferred to an E2 conjugating enzyme, where a thioester linkage with the catalytic cysteine of E2 is formed. Finally, the Ublp is covalently attached to the target protein via an isopeptide bond, with the assistance of an E3 ligase. [9]

Supplementary figures

Supplementary Figure 1

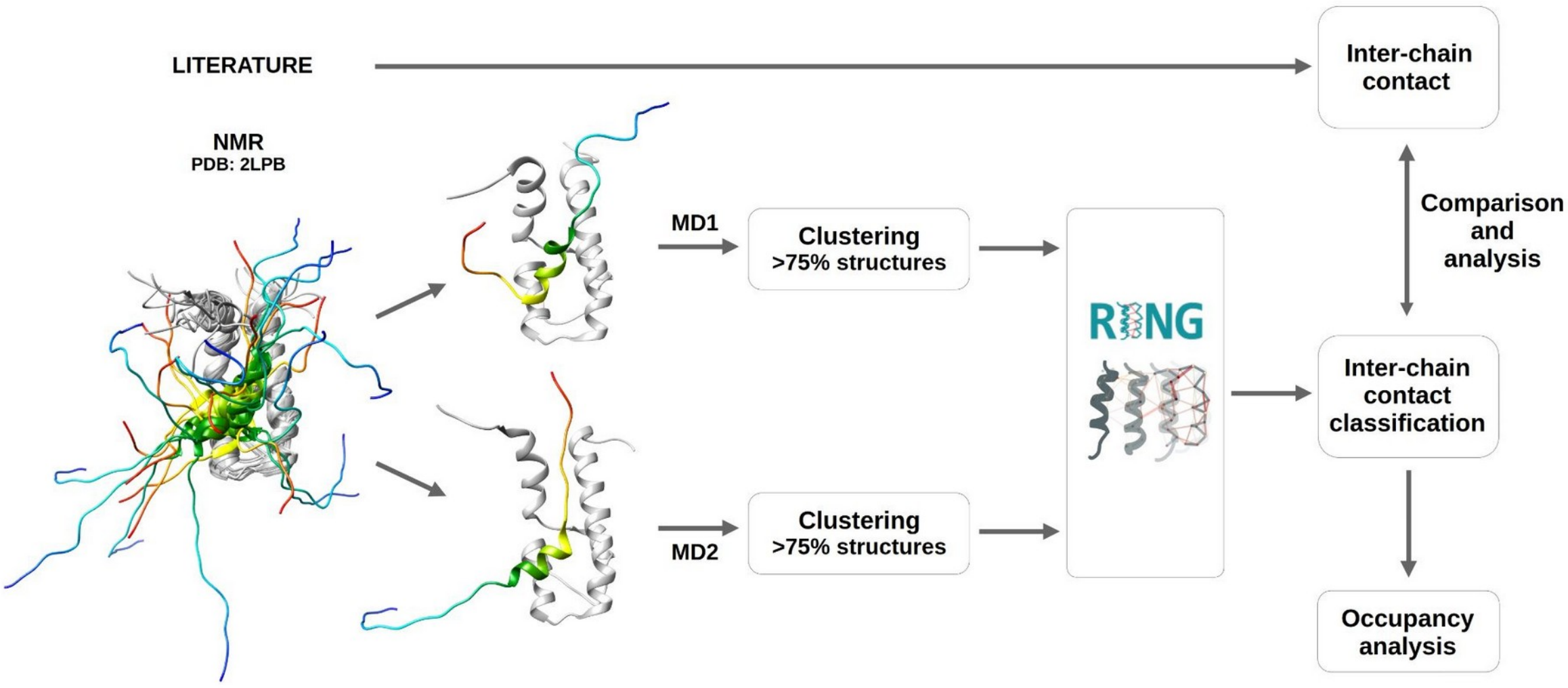

Supplementary Figure 1: workflow strategy using NMR, MD, Ring server [10] and costumed analysis.

Supplementary Figure 2

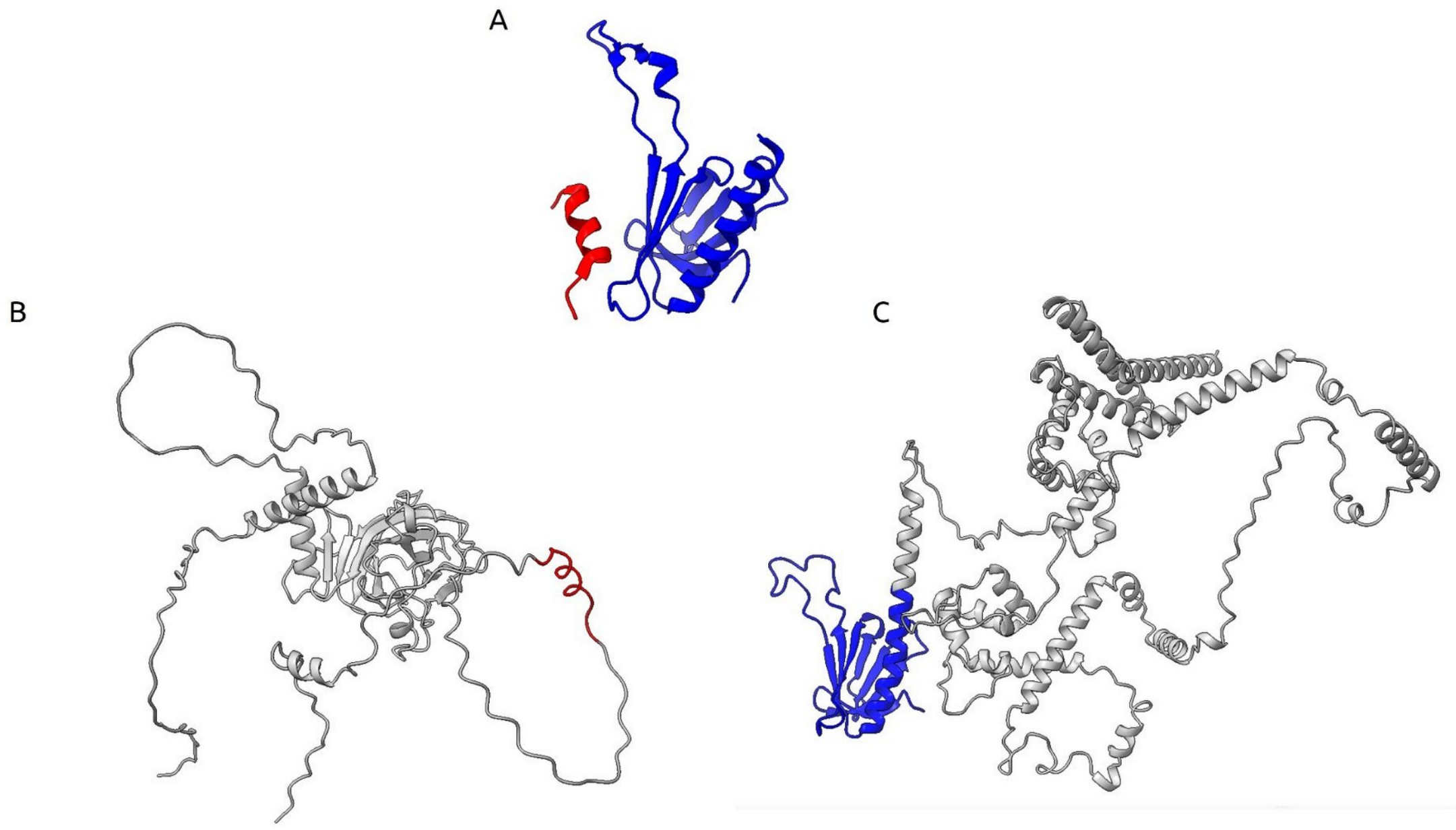

Supplementary Figure 2. TFB1-p53 complex. A. NMR structure of the TFB1–p53 complex (PDB: 2GS0). Ribbon representation of residues 1–115 of TFB1 (blue) and residues 45–58 of p53 (red). **B, C.** AlphaFold-predicted models of full-length p53 (B) and TFB1 (C), shown in

gray. Regions corresponding to the NMR-determined structure are highlighted in red for p53 and blue for TFB1 in each AlphaFold model.

Supplementary Figure 3:

A

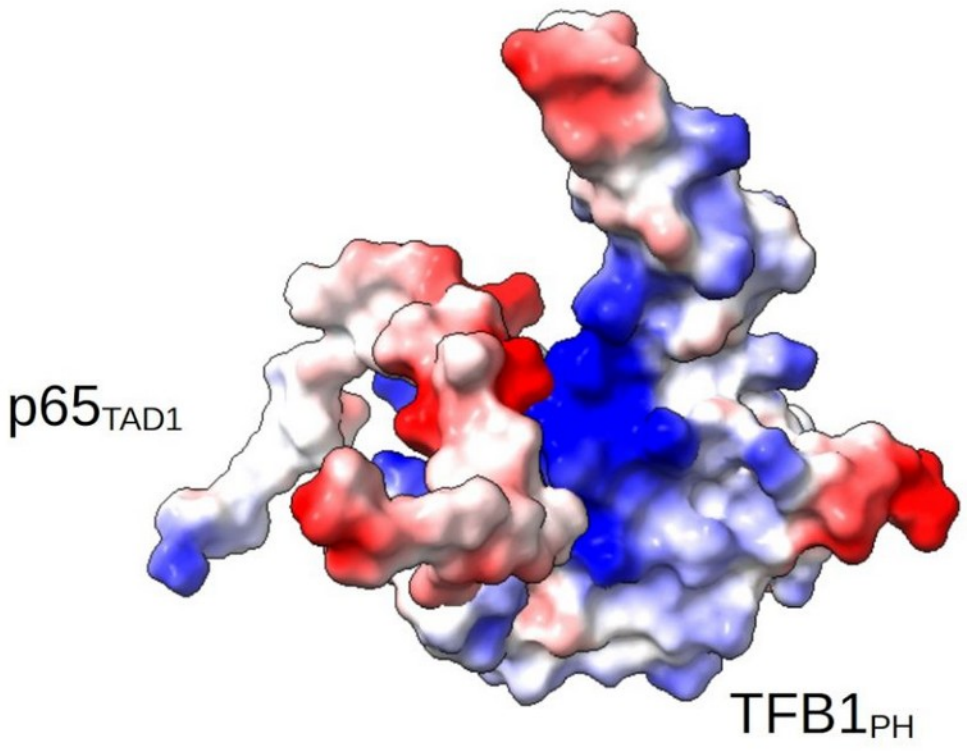

B

$p65_{TAD}$	$TFB1_{PH}$
D539	R61
D539	R86
**F542**	Q49
**F542**	A50
**F542**	T51
**F542**	M59
**F542**	R61
**F542**	M88
L546	Q49

C D

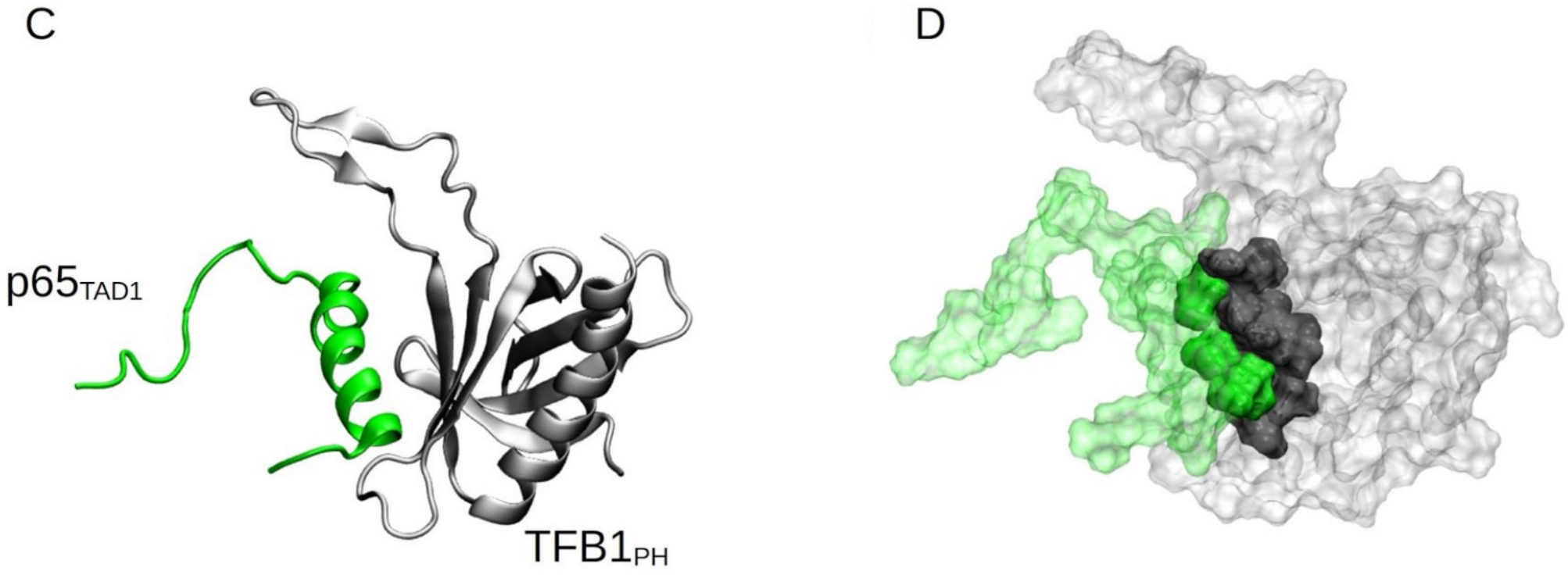

Supplementary Figure 3. $TFB1_{PH}$-$p65_{TAD}$ complex and core contacts. A. Model 1 of PDB 5URN is depicted in surface and colored by electrostatic potential. **B.** MD-detected core contacts, positive charged residues colored in blue, negative charged residues colored in red. **C.** Ribbon representation of $TFB1_{PH}$ in gray and $p65_{TAD1}$ colored in green, initial configuration of MD1. **D.** Surface representation of $TFB1_{PH}$ in gray and $p65_{TAD1}$ in green, initial configuration of MD1 depicted in transparent material) and core contacts depicted in opaque material.

Supplementary movies captions

Supplementary movie 1. Gal11$_{ABD1}$/GNC4$_{cAD}$ complex (PDB: 2LPB). MD1 (left) and MD2 (right), ribbon representation of GNC4 in green and Gal11 in gray; the core residues are depicted in sticks (F124 of GNC4 in yellow and W196 and L200 of Gal11 in purple); N-terminal amino acid is colored in blue, and C-terminal in red. N and C terminal segments (101-112 and 131-134 respectively) of GCN4 are shown in a transparent material to facilitate the visualization of the helix. Structures were aligned using the RMSD trajectory tool from VMD; both simulations were run separately, and are displayed side by side for simultaneous visualization. A trajectory smoothing window size of 5 was applied [11].

Supplementary movie 2. SF1$_{NTD}$/U2AF65$_{UHM}$ complex. Ribbon representation of SF1$_{NTD}$ in gray and U2AF65$_{UHM}$ in green; core residues are depicted in sticks. For HMGB1 core residues, located in a structured region, are colored in black, and those located in a disordered region are colored in light blue; for p53 core residues located in a structured region are colored in green, and those located in a disordered region are colored in yellow. U2AF65$_{UHM}$ (green), SF1$_{ULM}$ (orange) and SF1$_{HH}$ (gray), are depicted in ribbon; SF1$_{ULM}$ and SF1$_{HH}$ core residues are shown in sticks (orange and dark gray respectively). U2AF65$_{UHM}$-core residues involved in interactions with SF1$_{ULM}$ (cyan) and SF1$_{HH}$ (green) are shown, with the exception of residues E49 and K462, which are colored in red and blue, respectively. MD1 trajectory is presented in the movie. Structures were aligned using the RMSD trajectory tool from VMD; a trajectory smoothing window size of 5 was applied [4,11]**.**

Supplementary movie 3. CBPN$_{CBD}$/p53$_{TAD}$ complex (PDB: 2L14). Ribbon representation of CBPN in gray and p53 in green; core residues are depicted in sticks. For CBPN core residues located in a structured region are colored in black, and those located in a disordered region in light blue; for p53 core residues located in a structured region are colored in green, and those located in a disordered region in yellow. Structures were aligned using the RMSD trajectory tool from VMD; both simulations were run separately, and are displayed side by side for simultaneous visualization. A trajectory smoothing window size of 5 was applied [11]**.**

Supplementary movie 4. CBPN$_{CBD}$/p53$_{TAD}$ complex (PDB: 2L14). Ribbon representation of CBPN in gray and p53 in green; core residues are depicted in sticks. In blue R2105 from CBPN$_{CBD}$ colored and D49 from p53, in red. Structures were aligned using the RMSD trajectory tool from VMD; both simulations were run separately, and are displayed side by side for simultaneous visualization. A trajectory smoothing window size of 5 was applied [11]**.**

Supplementary movie 5. TFB1$_{PH}$/p65$_{TAD}$ complex (PDB: 5URN). Ribbon representation of TFB1$_{PH}$ in gray and p65$_{TAD}$ in green; core residues are depicted in sticks, hydrophobic residues (white), polar without charge residues (green), positively charged and negatively charged residues in blue and red respectively. Structures were aligned using the RMSD trajectory tool from VMD; both simulations were run separately, and are displayed side by side for simultaneous visualization. A trajectory smoothing window size of 5 was applied [11].

Supplementary movie 6. TFB1/p53 complex (PDB: 2GS0). Ribbon representation of TFB1 (gray) and p53 (green). N-terminal and C-terminal residues of p53 are shown in sticks in blue

and red respectively. Structures were aligned using the RMSD trajectory tool from VMD; both simulations were run separately, and are displayed side by side for simultaneous visualization. A trajectory smoothing window size of 5 was applied [11].

Supplementary movie 7. TFB1/p53 complex (PDB: 2GS0). Ribbon representation of TFB1 (gray) and p53 (green). Residue F54 of p53 is depicted in sticks and colored in magenta, residues Q49, A50, T51, M59 and R61 of TFB1 are depicted in sticks and colored in dark gray. Structures were aligned using the RMSD trajectory tool from VMD; both simulations were run separately, and are displayed side by side for simultaneous visualization. A trajectory smoothing window size of 5 was applied [11].

Supplementary movie 8. HMGB1/p53 complex (PDB: 2LY4). Ribbon representation of HMGB1 and p53. HMGB1 is colored in gray and p53 is colored by residue type (white non-polar residues, green polar residues, red and blue acidic and basic residues respectively). Hydrophobic residues of HMGB1 and p53 are shown in sticks and are colored in black and white respectively. Structures were aligned using the RMSD trajectory tool from VMD; both simulations were run separately, and are displayed side by side for simultaneous visualization. A trajectory smoothing window size of 10 was applied [11].

Supplementary movie 9. HMGB1/p53 complex (PDB: 2LY4). Ribbon representation of HMGB1 and p53. HMGB1 is colored in gray and p53 is colored by residue type (white non-polar residues, green polar residues, red and blue acidic and basic residues respectively). Positively charged residues of HMGB1 are shown in sticks and are colored in light blue, negatively charged residues of p53 are shown in sticks and colored in red. Structures were aligned using the RMSD trajectory tool from VMD; both simulations were run separately, and are displayed side by side for simultaneous visualization. A trajectory smoothing window size of 10 was applied [11].

Supplementary movie 10. Trajectory SUMO-1/PIASX-P complex (PDB: 2ASQ). Ribbon representation of the complex, and colored in green for MD1 and yellow for MD2. Structures were aligned using the RMSD trajectory tool from VMD; both simulations (run separately) were represented one on top of the other for a simultaneous analysis. PIASX-P is visualized as a small β-sheet detaching only at the beginning of the MD2. A trajectory smoothing window size of 5 was applied [11].

Supplementary movie 11. SAE2/UBC9 complex (PDB: 2PX9). Ribbon representation of the complex. The SAE2 cys domain position was aligned all over the trajectory to its initial position to keep it fixed; the secondary structures are colored in gray while disordered regions are colored with a rainbow pattern from blue (N-term) to red (C-term). In UBC9 the helical segments are colored in blue, orange and red and the beta sheets in yellow, and the disordered regions are depicted in magenta. Structures were aligned using the RMSD trajectory tool from VMD; both simulations were run separately, and are displayed side by side for simultaneous visualization. A trajectory smoothing window size of 5 was applied [11]

1. Brzovic PS, Heikaus CC, Kisselev L, Vernon R, Herbig E, Pacheco D, et al. Structure of the complex of the central activation domain of Gcn4 bound to the mediator co-activator domain 1 of Gal11/med15. Worldwide Protein Data Bank; 2012. doi:10.2210/pdb2lpb/pdb

2. Lee C, Martinez-Yamout MA, Dyson HJ, Wright PE. Structure of CBP nuclear coactivator binding domain in complex with p53 TAD. Worldwide Protein Data Bank; 2010. doi:10.2210/pdb2l14/pdb

3. Lee CW, Martinez-Yamout MA, Dyson HJ, Wright PE. Structure of the p53 transactivation domain in complex with the nuclear receptor coactivator binding domain of CREB binding protein. Biochemistry. 2010;49: 9964–9971.

4. Lecoq L, Raiola L, Chabot PR, Cyr N, Arseneault G, Legault P, et al. Structural characterization of interactions between transactivation domain 1 of the p65 subunit of NF-κB and transcription regulatory factors. Nucleic Acids Res. 2017;45: 5564–5576.

5. Madl T, Sattler M, Zhang Y, Bagdiul I, Kern T, Kang H, et al. Structure, phosphorylation and U2AF65 binding of the Nterminal Domain of splicing factor 1 during 3 splice site Recognition. Worldwide Protein Data Bank; 2013. doi:10.2210/pdb2m0g/pdb

6. Di Lello P, Jones TN, Nguyen BD, Legault P, Omichinski JG. NMR structure of the complex between the PH domain of the Tfb1 subunit from TFIIH and the activation domain of p53. Worldwide Protein Data Bank; 2006. doi:10.2210/pdb2gs0/pdb

7. Rowell JP, Simpson KL, Stott K, Watson M, Thomas JO. HMGB1-facilitated p53 DNA binding occurs via HMG-Box/p53 transactivation domain interaction, regulated by the acidic tail. Structure. 2012;20: 2014–2024.

8. Song J, Zhang Z, Hu W, Chen Y. Solution structure of SUMO-1 in complex with a SUMO-binding motif (SBM). Worldwide Protein Data Bank; 2005. doi:10.2210/pdb2asq/pdb

9. Wang JH, Hu WD, Cai S, Lee B, Song J, Chen Y. The intrinsic affinity between E2 and the Cys domain of E1 in Ubiquitin-like modifications. Worldwide Protein Data Bank; 2007. doi:10.2210/pdb2px9/pdb

10. Del Conte A, Camagni GF, Clementel D, Minervini G, Monzon AM, Ferrari C, et al. RING 4.0: faster residue interaction networks with novel interaction types across over 35,000 different chemical structures. Nucleic Acids Res. 2024 Jul 5;52(W1):W306–12. http://dx.doi.org/10.1093/nar/gkae337

11. Humphrey W, Dalke A, Schulten K. VMD: visual molecular dynamics. J Mol Graph. 1996;14: 33–8, 27–8.

Appendix 1: Temporary evolution of key contacts by complex.

Occupancy analysis of intermolecular contacts. For each complex, the contact time series for MD1 and MD2 are shown in green and blue, respectively. The calculated occupancy values and corresponding contact classification are summarized in the table.

Gal11$_{ABD1}$-Gnc4$_{cAD}$ complex (PDB: 2LPB)

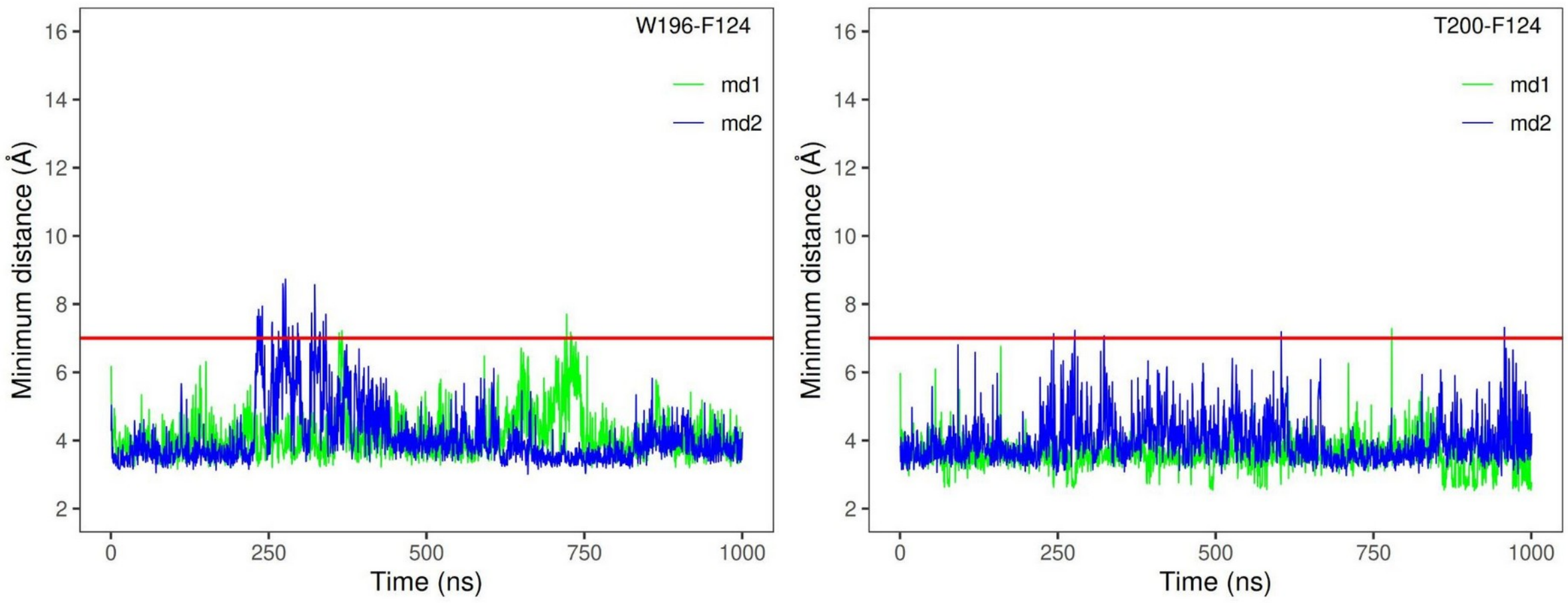

Contact	Occupancy (md1)	Level (md1)	Occupancy (md2)	Level (md2)
W196-F124	1.00	Total	0.98	Very high
T200-F124	1.00	Total	1.00	Total

SF1$_{NTD}$/U2AF65$_{UHM}$ complex (PDB: 2M0G)

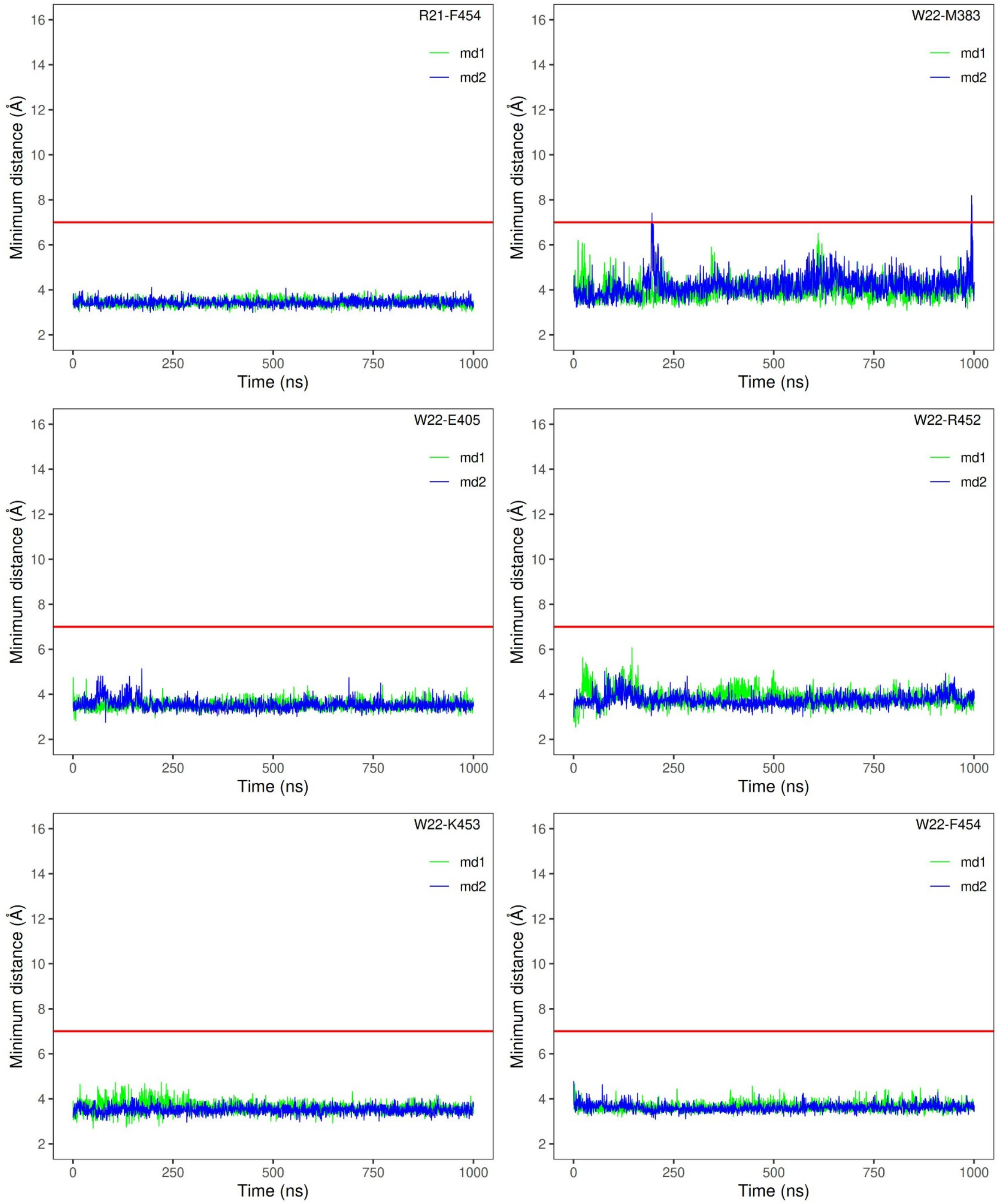

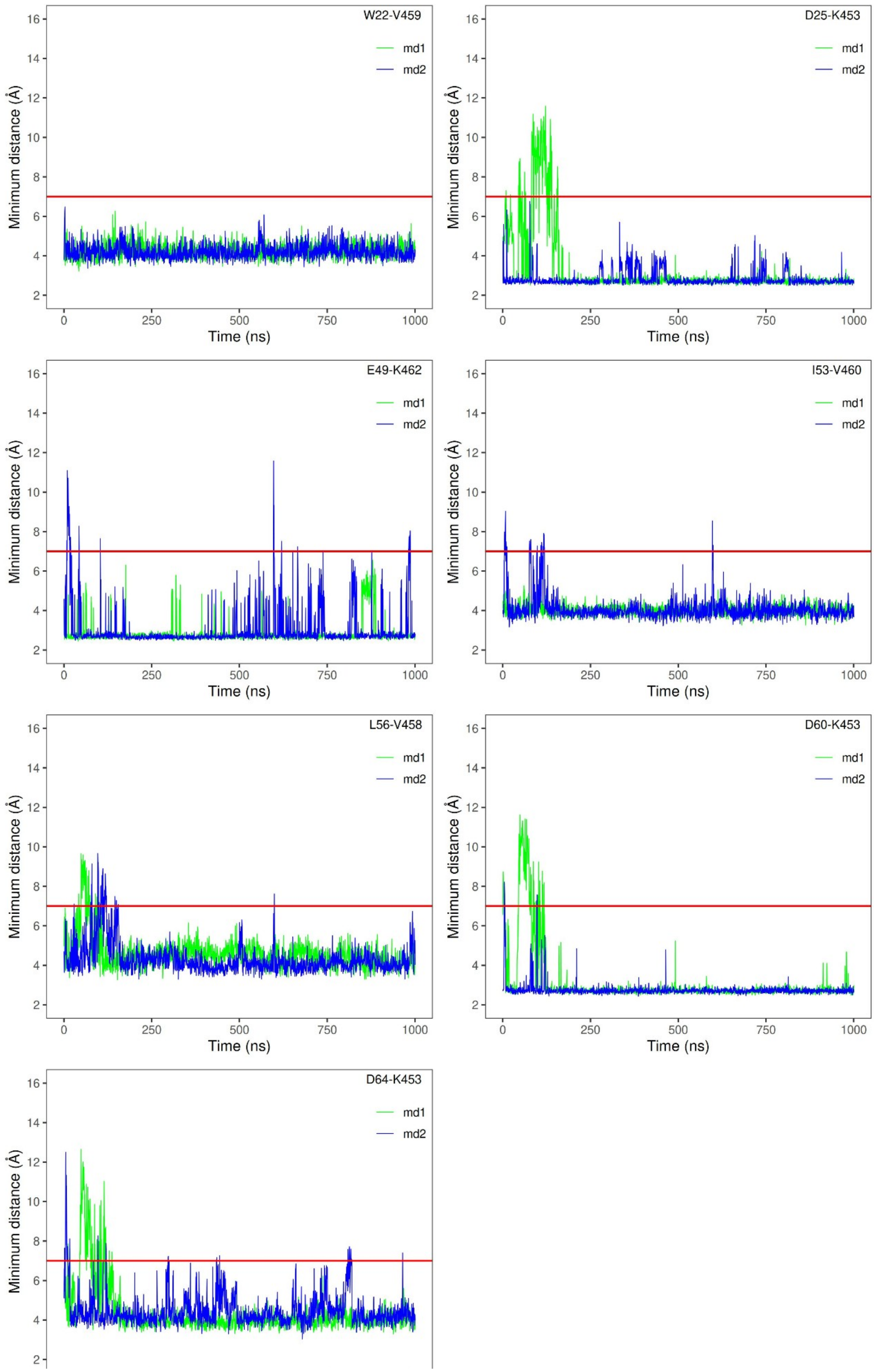
W22-V459
D25-K453
E49-K462
I53-V460
L56-V458
D60-K453
D64-K453
md1
md2
Minimum distance (Å)
Time (ns)

Contact	Occupancy (md1)	Level (md1)	Occupancy (md2)	Level (md2)
R21-F454	1.00	Total	1.00	Total
W22-M383	1.00	Total	1.00	Total
W22-E405	1.00	Total	1.00	Total
W22-R452	1.00	Total	1.00	Total
W22-K453	1.00	Total	1.00	Total
W22-F454	1.00	Total	1.00	Total
W22-V459	1.00	Total	1.00	Total
D25-K453	0.94	Very high	1.00	Total
E49-K462	1.00	Total	0.98	Very high
I53-V460	1.00	Total	0.99	Very high
L56-V458	0.98	Very high	0.98	Very high
D60-K453	0.96	Very high	1.00	Total
D64-K453	0.95	Very high	0.98	Very high

$CBPN_{CBD}$-$p53_{TAD}$ complex (PDB: 2L14)

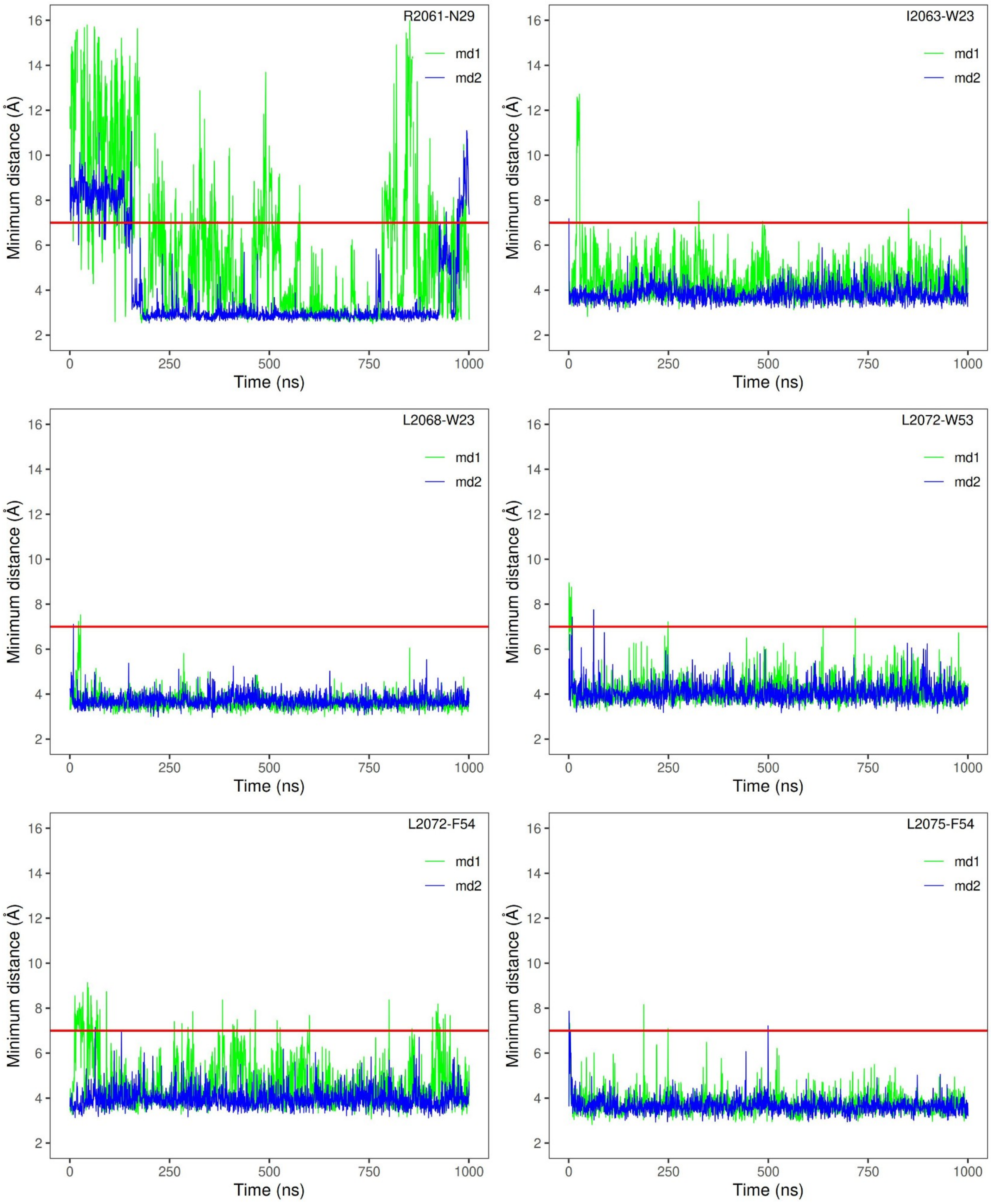

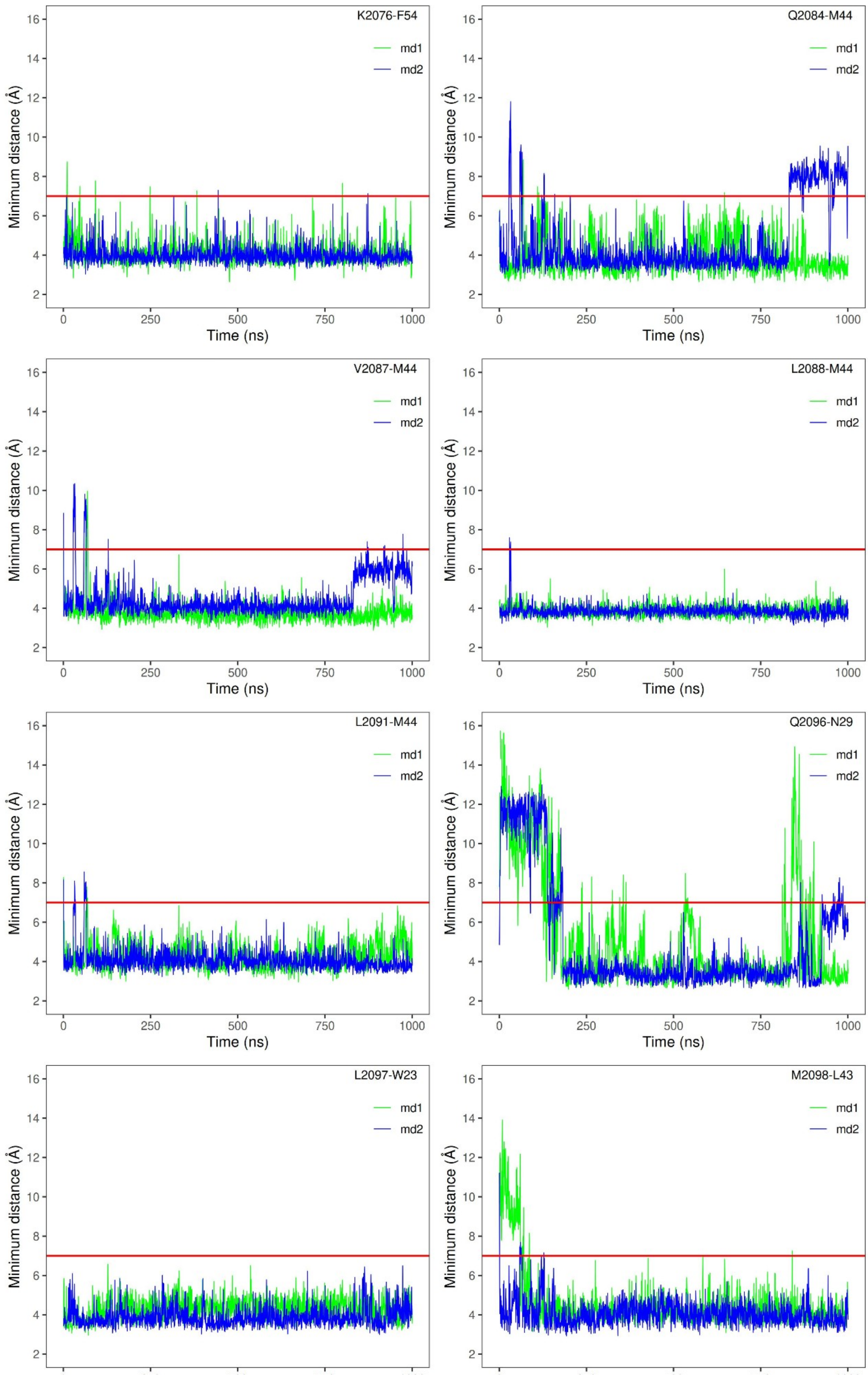

K2076-F54
Q2084-M44
V2087-M44
L2088-M44
L2091-M44
Q2096-N29
L2097-W23
M2098-L43
md1
md2
Minimum distance (Å)
Time (ns)
2
4
6
8
10
12
14
16
0
250
500
750
1000

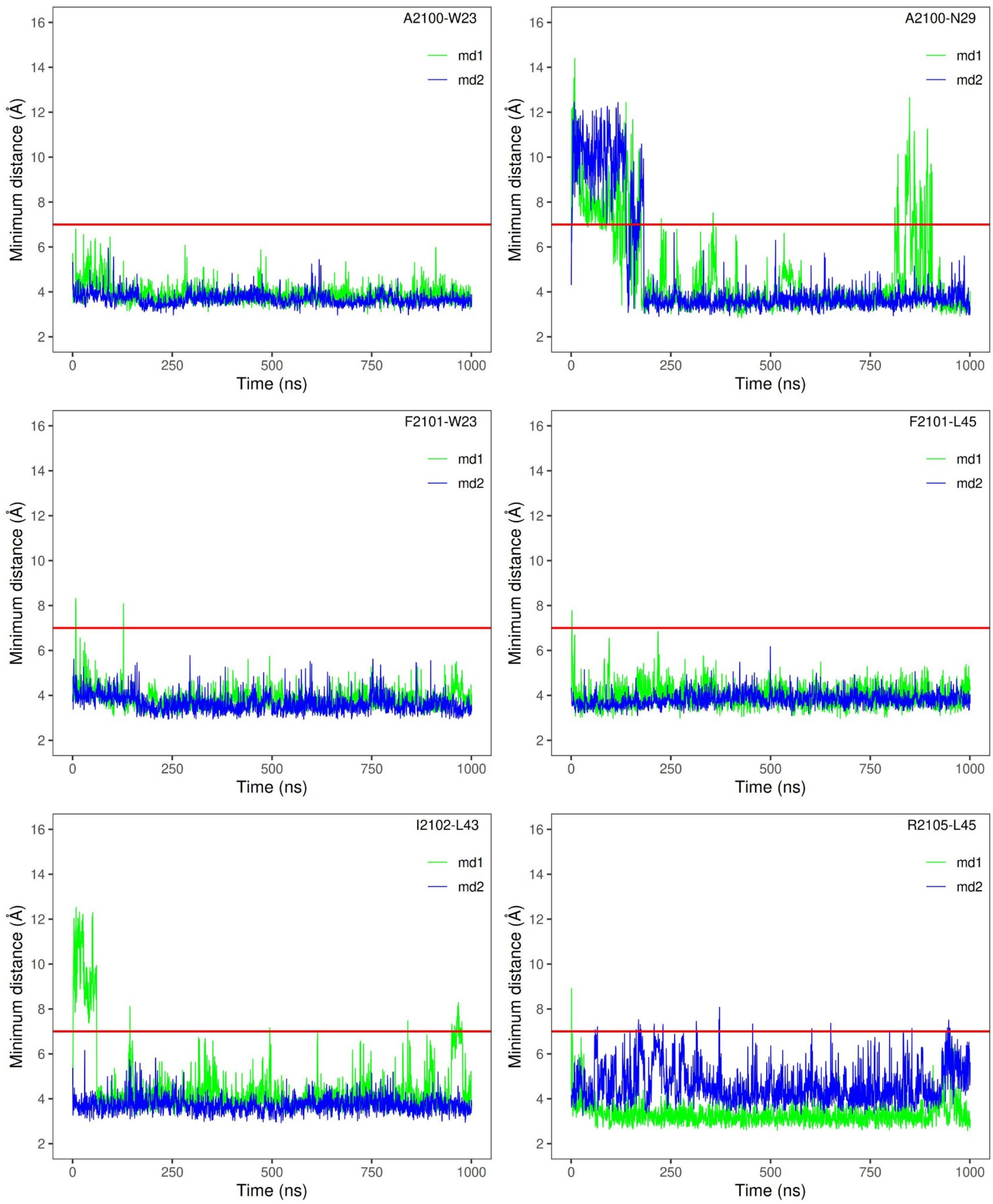
A2100-W23
A2100-N29
F2101-W23
F2101-L45
I2102-L43
R2105-L45
md1
md2
Minimum distance (Å)
Time (ns)

Contact	Occupancy (md1)	Level (md1)	Occupancy (md2)	Level (md2)
R2061-N29	0.71	Low	0.83	High
I2063-W23	0.99	Very high	1.00	Total
L2068-W23	1.00	Total	1.00	Total
L2072-W53	0.99	Very high	1.00	Total
L2072-F54	0.96	Very high	1.00	Total
L2075-F54	1.00	Total	1.00	Total
K2076-F54	1.00	Total	1.00	Total
Q2084-M44	1.00	Total	0.83	High
V2087-M44	0.99	Very high	0.98	Very high
L2088-M44	1.00	Total	1.00	Total
L2091-M44	1.00	Total	0.99	Very high
Q2096-N29	0.81	High	0.83	High
L2097-W23	1.00	Total	1.00	Total
M2098-L43	0.93	Very high	0.99	Very high
A2100-W23	1.00	Total	1.00	Total
A2100-N29	0.83	High	0.84	High
F2101-W23	1.00	Total	1.00	Total
F2101-L45	1.00	Total	1.00	Total
I2102-L43	0.93	Very high	1.00	Total
R2105-L45	1.00	Total	0.99	Very high

TFB1$_{PH}$-p65$_{TAD}$ complex (PDB: 5URN)

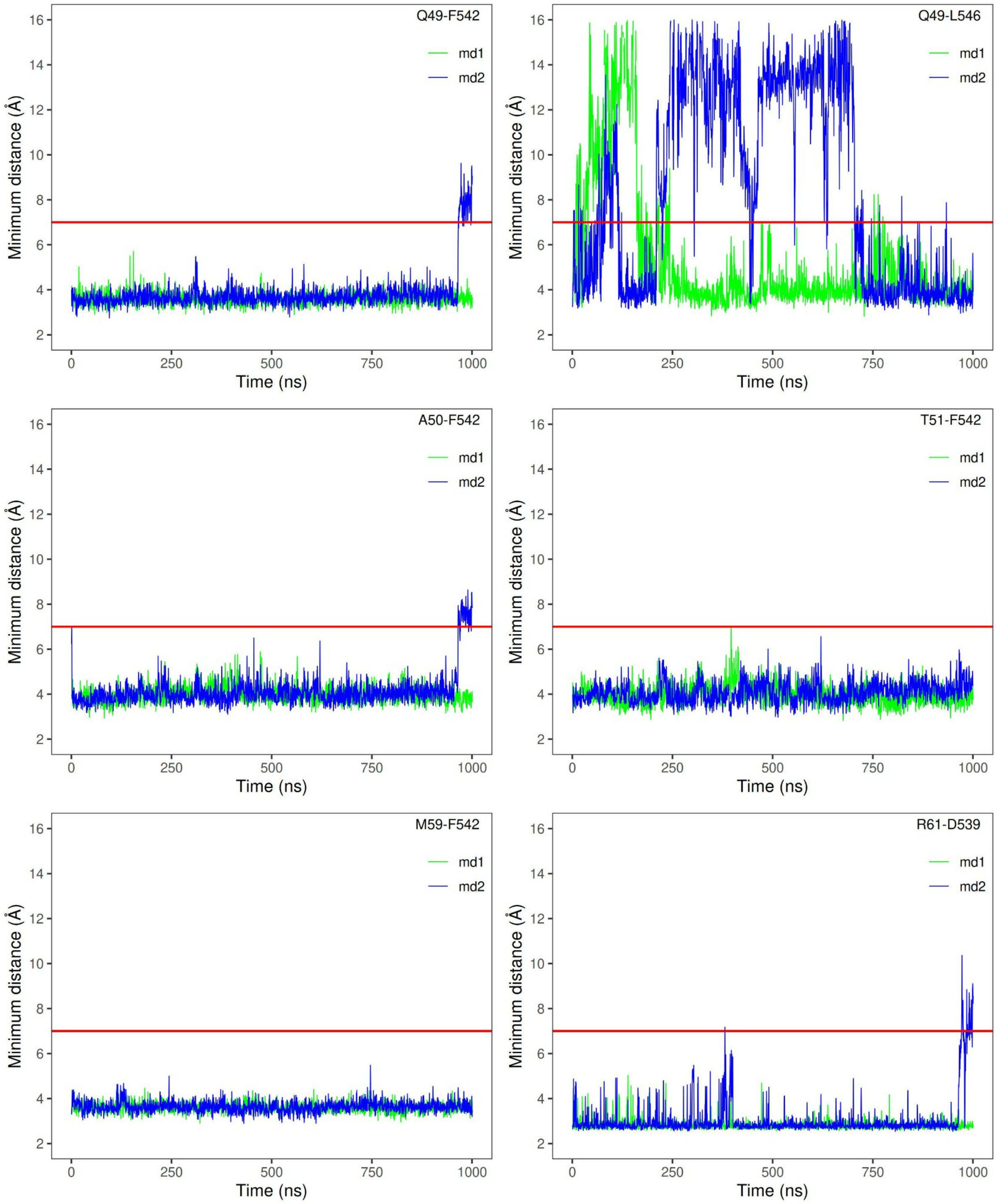

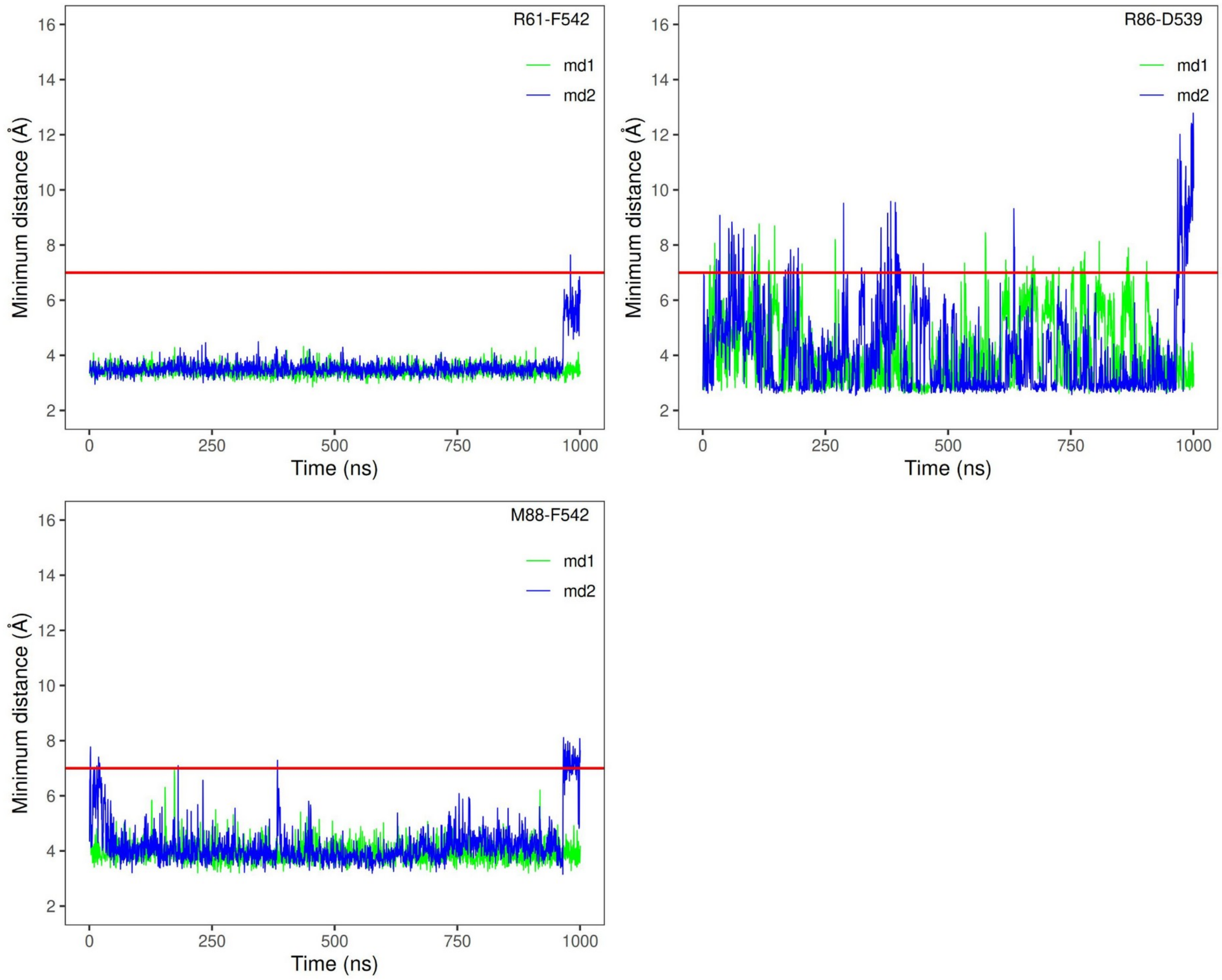

Contact	Occupancy (md1)	Level (md1)	Occupancy (md2)	Level (md2)
Q49-F542	1.00	Total	0.97	Very high
Q49-L546	0.84	High	0.47	Low
A50-F542	1.00	Total	0.97	Very high
T51-F542	1.00	Total	1.00	Total
M59-F542	1.00	Total	1.00	Total
R61-D539	1.00	Total	0.98	Very high
R61-F542	1.00	Total	1.00	Total
R86-D539	0.98	Very high	0.94	Very high
M88-F542	1.00	Total	0.97	Very high

$TFB1_{PH}$/$p53_{TAD}$ complex (PDB: 2GS0)

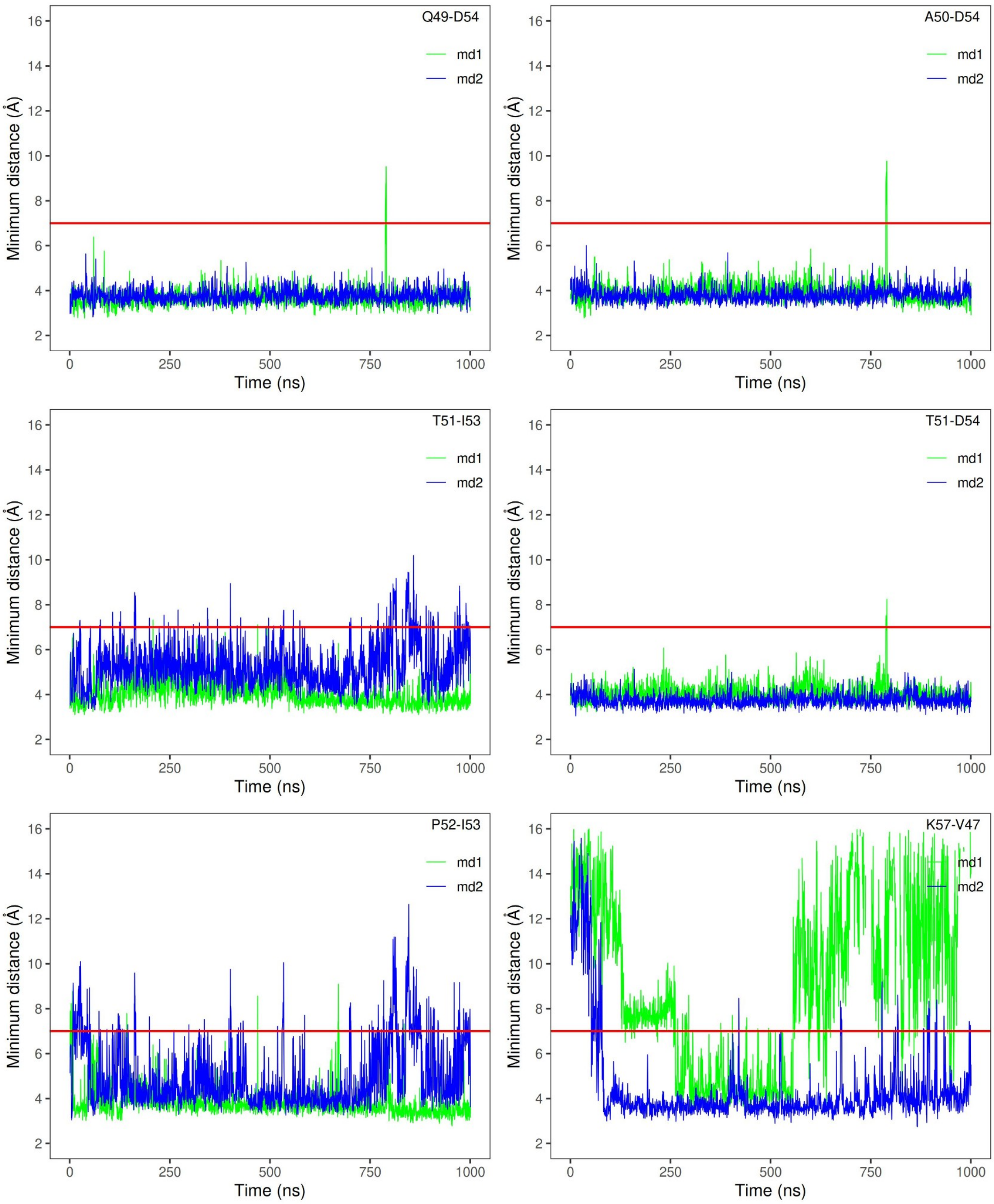
Q49-D54
A50-D54
T51-I53
T51-D54
P52-I53
K57-V47
md1
md2
Minimum distance (Å)
Time (ns)
0
250
500
750
1000
2
4
6
8
10
12
14
16

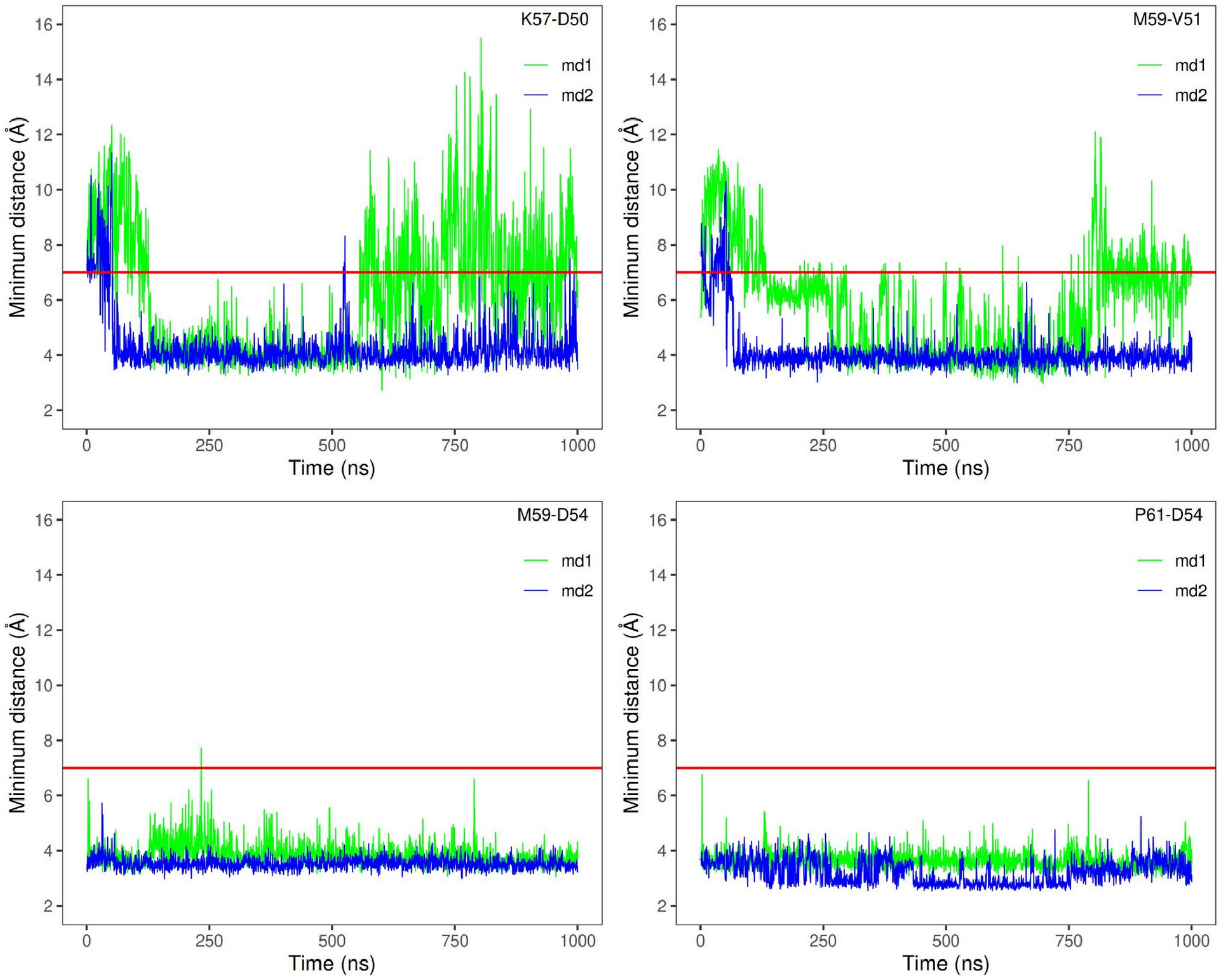

Contact	Occupancy (md1)	Level (md1)	Occupancy (md2)	Level (md2)
Q49-D54	1.00	Total	1.00	Total
A50-D54	1.00	Total	1.00	Total
T51-I53	1.00	Total	0.93	Very high
T51-D54	1.00	Total	1.00	Total
P52-I53	0.99	Very high	0.88	Very high
K57-V47	0.32	Low	0.92	Very high
K57-D50	0.66	Low	0.96	Very high
M59-V51	0.78	High	0.97	Very high
M59-D54	1.00	Total	1.00	Total
P61-D54	1.00	Total	1.00	Total

SUMO-1/PIASX-P complex (PDB: 2ASQ)

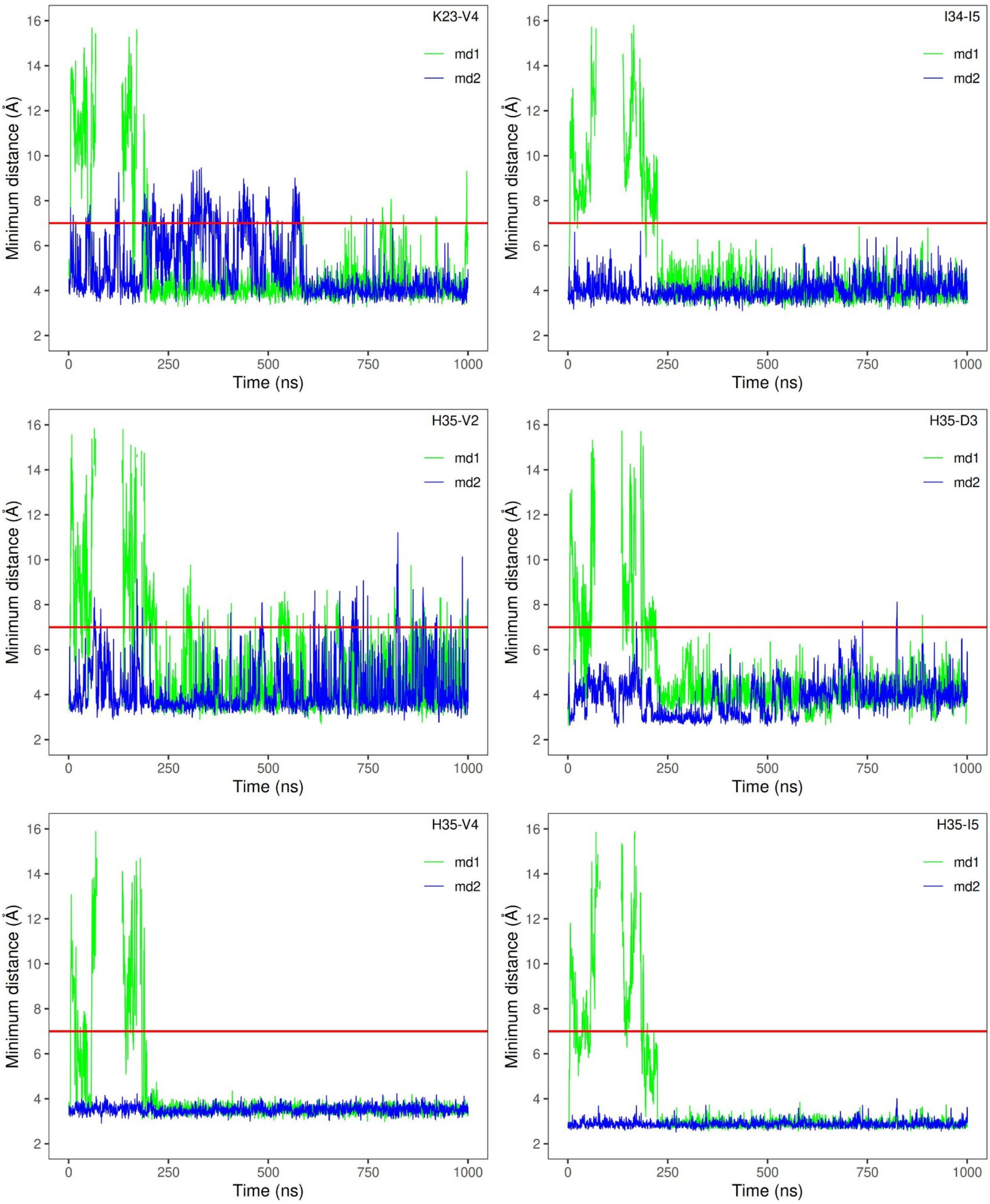

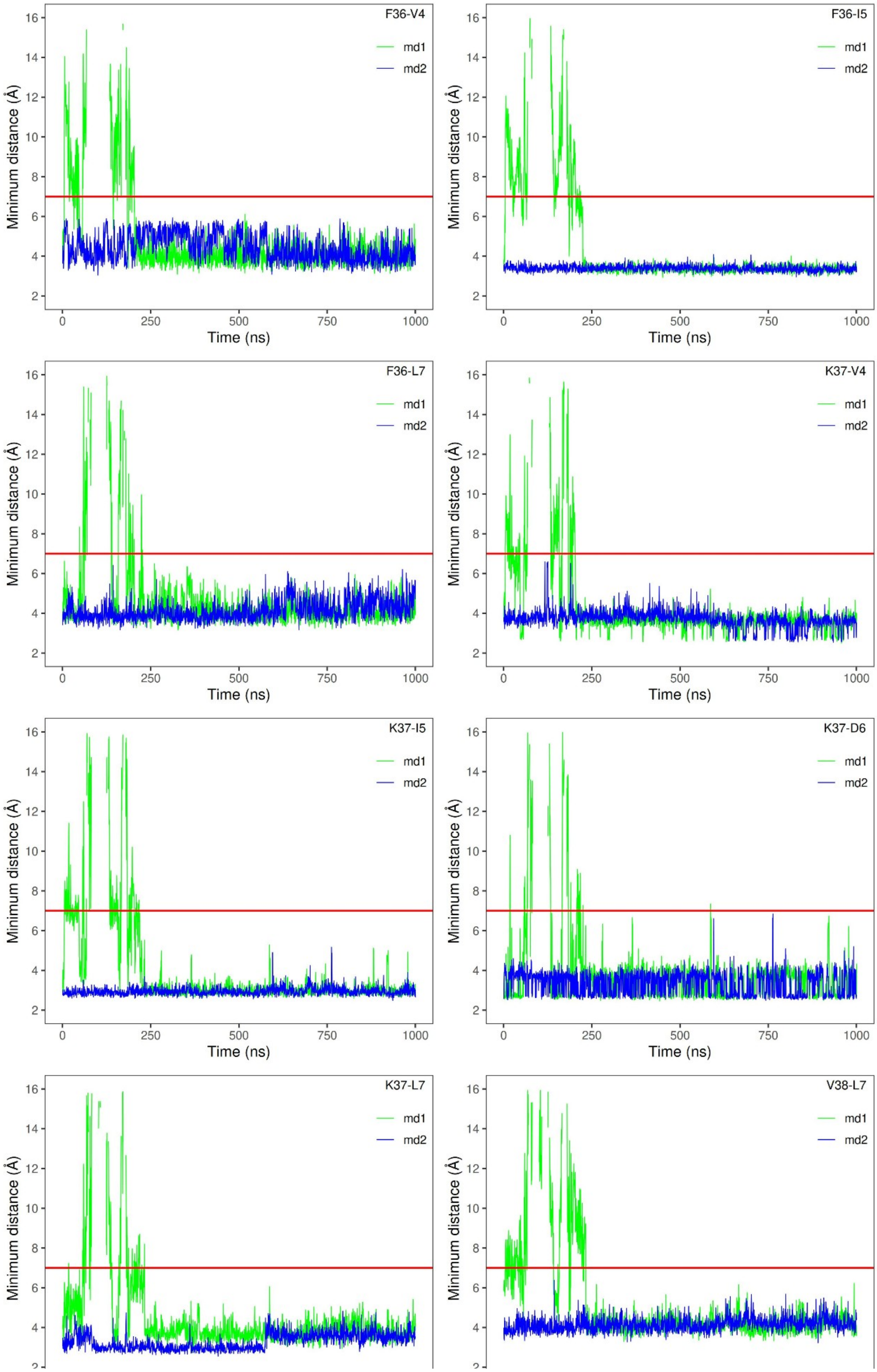

F36-V4
F36-I5
F36-L7
K37-V4
K37-I5
K37-D6
K37-L7
V38-L7
md1
md2
Minimum distance (Å)
Time (ns)
0
250
500
750
1000
2
4
6
8
10
12
14
16

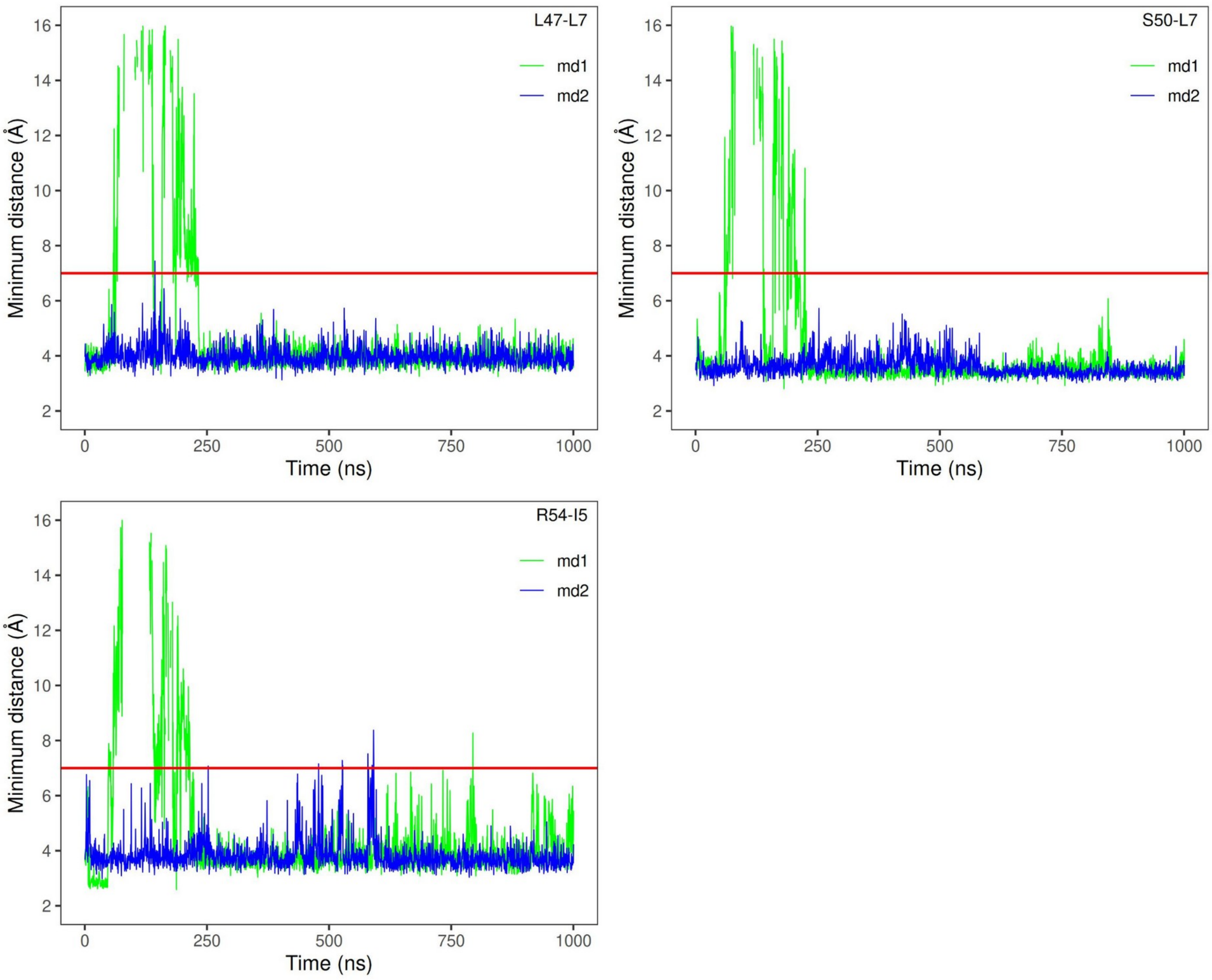
L47-L7
md1
md2
Minimum distance (Å)
Time (ns)
S50-L7
md1
md2
Minimum distance (Å)
Time (ns)
R54-I5
md1
md2
Minimum distance (Å)
Time (ns)

Contact	Occupancy (md1)	Level (md1)	Occupancy (md2)	Level (md2)
K23-V4	0.81	High	0.87	Very high
I34-I5	0.78	High	1.00	Total
H35-V2	0.75	High	0.97	Very high
H35-D3	0.82	High	1.00	Total
H35-V4	0.86	Very high	1.00	Total
H35-I5	0.84	High	1.00	Total
F36-V4	0.82	High	1.00	Total
F36-I5	0.81	High	1.00	Total
F36-L7	0.89	Very high	1.00	Total
K37-V4	0.86	Very high	1.00	Total
K37-I5	0.87	Very high	1.00	Total
K37-D6	0.91	Very high	1.00	Total
K37-L7	0.89	Very high	1.00	Total
V38-L7	0.82	High	1.00	Total
L47-L7	0.85	Very high	1.00	Total
S50-L7	0.89	Very high	1.00	Total
R54-I5	0.86	Very high	1.00	Total
